# Ultrastructural and Functional Analysis of a Novel Extra-Axonemal Structure in Parasitic Trichomonads

**DOI:** 10.3389/fcimb.2021.757185

**Published:** 2021-11-09

**Authors:** Veronica M. Coceres, Lucrecia S. Iriarte, Abigail Miranda-Magalhães, Thiago André Santos de Andrade, Natalia de Miguel, Antonio Pereira-Neves

**Affiliations:** ^1^ Laboratorio de Parásitos Anaerobios, Instituto Tecnológico Chascomús (INTECH), Consejo Nacional de Investigaciones Científicas y Técnicas - Universidad Nacional de General San Martín (CONICET-UNSAM), Chascomús, Argentina; ^2^ Departamento de Microbiologia, Instituto Aggeu Magalhães, FIOCRUZ, Recife, Brazil; ^3^ Departamento de Imunologia, Instituto Aggeu Magalhães, FIOCRUZ, Recife, Brazil

**Keywords:** *Trichomonas vaginalis*, *Tritrichomonas foetus*, flagella, electron microscopy, parasite–host cell interaction, cell attachment, VPS32

## Abstract

*Trichomonas vaginalis* and *Tritrichomonas foetus* are extracellular flagellated parasites that inhabit humans and other mammals, respectively. In addition to motility, flagella act in a variety of biological processes in different cell types, and extra-axonemal structures (EASs) have been described as fibrillar structures that provide mechanical support and act as metabolic, homeostatic, and sensory platforms in many organisms. It has been assumed that *T. vaginalis* and *T. foetus* do not have EASs. However, here, we used complementary electron microscopy techniques to reveal the ultrastructure of EASs in both parasites. Such EASs are thin filaments (3–5 nm diameter) running longitudinally along the axonemes and surrounded by the flagellar membrane, forming prominent flagellar swellings. We observed that the formation of EAS increases after parasite adhesion on the host cells, fibronectin, and precationized surfaces. A high number of rosettes, clusters of intramembrane particles that have been proposed as sensorial structures, and microvesicles protruding from the membrane were observed in the EASs. Our observations demonstrate that *T. vaginalis* and *T. foetus* can connect to themselves by EASs present in flagella. The protein VPS32, a member of the ESCRT-III complex crucial for diverse membrane remodeling events, the pinching off and release of microvesicles, was found in the surface as well as in microvesicles protruding from EASs. Moreover, we demonstrated that the formation of EAS also increases in parasites overexpressing VPS32 and that *T. vaginalis*-VPS32 parasites showed greater motility in semisolid agar. These results provide valuable data about the role of the flagellar EASs in the cell-to-cell communication and pathogenesis of these extracellular parasites.

## 1 Introduction

The eukaryotic flagella are highly conserved microtubule-based organelles that extend from the cell surface. These structures, beyond being essential for cell locomotion and movement of fluids across the tissues and cells, are signaling platforms that receive and send information to drive cellular responses (Carter and Blacque, 2019; Akella et al., 2020). These functions are crucial for health, development, and reproduction processes in most eukaryotes, including humans (Anvarian et al., 2019; Wan and Jekely, 2020). In addition to cell movement (Imhof et al., 2019) and sensory functions (Maric et al., 2010), a variety of microorganisms employ flagella to control feeding (Dolger et al., 2017), mating (Fussy et al., 2017), cytokinesis (Ralston et al., 2006; Hardin et al., 2017), cell morphogenesis (Vaughan, 2010), cell communication (Szempruch et al., 2016), and cell adhesion (Frolov et al., 2018). Among these microorganisms, there are important human and veterinary parasitic protists, i.e., trichomonads, trypanosomatids, diplomonads, and apicomplexa, that exert a devastating economic burden on global healthcare systems and agriculture (Kruger and Engstler, 2015).

The trichomonads (Metamonada, Parabasalia) *Trichomonas vaginalis* and *Tritrichomonas foetus* are extracellular parasites that inhabit humans and other mammals, respectively. *Trichomonas vaginalis* is responsible for trichomoniasis, the most common non-viral sexually transmitted infection in men and women (WHO, 2018). Most infected people are asymptomatic, but when symptoms do occur, they can range from mild irritation to severe inflammation in various regions of the reproductive tract (Van Gerwen and Muzny, 2019). *Trichomonas vaginalis* is also associated with pelvic inflammatory disease, pregnancy complications, preterm birth, and infertility (Kissinger, 2015; Meites et al., 2015), as well as increased risk to HIV (McClelland et al., 2007; Van Der Pol et al., 2008), papillomavirus infection, and cervical or prostate cancer (Gander et al., 2009; Stark et al., 2009; Sutcliffe et al., 2009; Twu et al., 2014). *Tritrichomonas foetus* is a widespread pathogen that colonizes the reproductive tract of cattle and the large intestine of cats, leading to bovine and feline tritrichomonosis, respectively. Bovine tritrichomonosis is a venereal infection that causes significant economic losses in beef and dairy farming due to early embryonic death, abortion and infertility, or culling of parasite carriers (Martin-Gomez et al., 1998; Mardones et al., 2008). Feline tritrichomonosis causes chronic diarrhea in cats (Gookin et al., 2017). *Tritrichomonas foetus* also lives as a commensal in the nasal and gastrointestinal mucosa of pigs (Dabrowska et al., 2020).

In each trichomonads genus, the flagella vary in number and size: *T. vaginalis* and *T. foetus* have five and four flagella, respectively (Benchimol, 2004). Like most eukaryotes, the structural basis of the trichomonads motile flagella is the canonical “9 + 2” microtubular axoneme surrounded by plasma membrane (Benchimol, 2004). In both species, the plasma membrane of the anterior flagella has rosette-like formations that have been proposed as sensorial structures (Benchimol et al., 1982; Honigberg et al., 1984). Based on this, some authors have suggested that the flagella could be involved in migration and sensory reception in trichomonads during adherence to host tissue and amoeboid morphogenesis (de Miguel et al., 2012; Kusdian et al., 2013; Lenaghan et al., 2014). However, the flagellar role during parasite cell adhesion, amoeboid transformation, and cell-to-cell communication is still poorly understood.

In other organisms, flagella can send information *via* ectosomes (also called microvesicles), a type of extracellular vesicle that protrudes and sheds from the cell surface (Wang and Barr, 2018). In *Trypanosoma*, these ectosomes can transfer virulence factors from one parasite to the other contributing to the pathogenesis (Szempruch et al., 2016). In this sense, our group recently reported that *T. vaginalis* releases flagellar ectosomes that might have an important role in cell communication (Nievas et al., 2018). Proteins from the endosomal sorting complex required for transport (ESCRT) machinery are involved in flagellar ectosome release in protists. Specifically, ESCRT-III proteins may play a central role in promoting ectosome budding from the flagellum membrane (Long et al., 2016). However, the localization and possible functions of ESCRT proteins in the trichomonad flagella have not been determined yet.

In addition to axoneme and ectosomes, the assembly of extra-axonemal structures (EASs) occurs in many organisms ranging from mammals and insects (Zhao et al., 2018; Miao et al., 2019) to protists, e.g., euglenozoa, dinoflagellates, and *Giardia* (Portman and Gull, 2010; Maia-Brigagao et al., 2013; Moran et al., 2014). EASs are evolutionarily convergent, highly organized fibrillar structures that provide mechanical support and act as metabolic, homeostatic, and sensory platforms for the regulation of flagellar beating (Portman and Gull, 2010; Moran et al., 2014). Depending on the cell type, EASs can be symmetrically or asymmetrically arranged around the axoneme and they can run along almost the entire length or only a portion of the flagellum (Portman and Gull, 2010). In protists, the paraflagellar rod (PFR), which is seen in trypanosomatids, is the best characterized EAS. PFR is required for motility, parasite attachment to host cells, morphogenesis, and cell division (Portman and Gull, 2010). Although EASs, formed by thin filaments, have been described in some trichomonads and related parabasalid species (Mattern et al., 1973; Brugerolle et al., 1994; Brugerolle, 1999; Brugerolle, 2005), there are no reports on the existence and role of EASs in *T. vaginalis* and *T. foetus.* In this work, using a detailed ultrastructural analysis, we identified the presence of EASs forming prominent flagellar swellings in *T. vaginalis* and *T. foetus*. Interestingly, we found an increase of EAS formation after parasite adhesion on the host cells, fibronectin, and precationized surfaces. A high number of rosettes and microvesicles protruding from the membrane can be found in the EAS. We also observed that parasites can connect to each other by EASs. Finally, we found that overexpression of a member of the ESCRT-III complex localized at the flagellar swelling, named VPS32, increased EAS formation and parasite motility in semisolid medium. Our data highlight a role for the EAS in the cell-to-cell communication and pathogenesis in *T. vaginalis* and *T. foetus.*

## 2 Materials and Methods

### 2.1 Parasite Culture

The *T. vaginalis* strains B7RC2 (parental, ATCC 50167), Jt, and FMV1 (Midlej and Benchimol, 2010) and *T. foetus* K (parental) and CC09-1s strains (Pereira-Neves et al., 2014) were cultured in Diamond’s Trypticase-yeast extract-maltose (TYM) medium supplemented with 10% bovine serum and 10 U/ml penicillin/10 μg/ml streptomycin (Invitrogen). Parasites were grown at 37°C and passaged daily, and 100 μg/ml G418 (Invitrogen) was added to the culture of the TvEpNeo/TvVPS32-HA and TfEpNeo/TfVPS32-HA transfectants.

### 2.2 Plasmid Construction and Exogenous Protein Expression in Trichomonads

The TvVPS32 construct was generated using primers with *Nde*I and *Kpn*I restriction sites engineered into the 5′- and 3′-primers, respectively. Polymerase chain reaction fragments were generated using standard procedures, and the resulting fragments were then cloned into the Master-Neo-(HA)_2_ plasmid to generate constructs to transfect into *T. vaginalis* and *T. foetus*. Electroporation of *T. vaginalis* G3 strain was carried out as described previously (Delgadillo et al., 1997), with 50 μg of circular plasmid DNA. Transfectants were selected with 100 mg/ml G418 (Sigma). The TfVPS32 construct was generated and transfected into *T. foetus* K as previously described (Iriarte et al., 2018).

### 2.3 Scanning Electron Microscopy

Cells were washed with phosphate buffered saline (PBS) and fixed in 2.5% glutaraldehyde in 0.1 M cacodylate buffer, pH 7.2. The cells were then post-fixed for 15 min in 1% OsO_4_, dehydrated in ethanol, and critical point-dried with liquid CO_2_. The dried cells were coated with gold–palladium to a thickness of 25 nm and then observed with a Jeol JSM-5600 scanning electron microscope, operating at 15 kV.

### 2.4 Transmission Electron Microscopy

#### 2.4.1 Routine Preparation

The parasites were washed with PBS and fixed in 2.5% glutaraldehyde in 0.1 M cacodylate buffer, pH 7.2. The cells were then post-fixed for 30 min in 1% OsO_4_, dehydrated in acetone, and embedded in Epon (Polybed 812). Ultrathin sections were harvested on 300 mesh copper grids, stained with 5% uranyl acetate and 1% lead citrate, and observed with a FEI Tecnai Spirit transmission electron microscope. The images were randomly acquired with a CCD camera system (MegaView G2, Olympus, Germany).

#### 2.4.2 Negative Staining

Parasites were settled onto positively charged Alcian blue-coated carbon film nickel grids (Labhart and Koller, 1981) for 30 min at 37°C. Next, cells were fixed in 2.5% glutaraldehyde in PEME (100 mM PIPES pH 6.9, 1 mM MgSO_4_, 2 mM EGTA, 0.1 mM EDTA) for 1 h at room temperature. To better visualize the axoneme and EAS, parasites were permeabilized with 1% Triton X-100 for 10 min, washed with water, and negatively stained with 1% aurothioglucose (UPS Reference Standard) in water for 5 s. Alternatively, non-permeabilized cells were stained with 2% uranyl acetate in water for 10 s in order to visualize the flagellar rosettes. The grids were then air-dried and observed as described above.

#### 2.4.3 Immunogold

Parasites were settled onto nickel grids as mentioned above, followed by fixation with 4% paraformaldehyde and 0.5% glutaraldehyde in PEME for 1 h at room temperature. After washes in PEME, the grids were incubated with 1% Triton X-100 in PEME for 10 min and quenched in 50 mM ammonium chloride, 3% and 1% BSA, and 0.2% Tween-20 in PBS (pH 8.0). Next, the grids were incubated with anti-HA tag antibody (Invitrogen, 5B1D10), 10× diluted in 1% BSA in PBS for 3 h at room temperature. The grids were washed with 1% BSA in PBS and labeled for 60 min with 10 nm gold-labeled goat anti-mouse IgG (BB International, UK), 100× diluted in 1% BSA in PBS, at room temperature. Samples were washed with PEME and water, negatively stained, and observed as mentioned above. As negative control, the primary antibodies were omitted, and the samples were incubated with the gold-labeled goat anti-mouse antibody only. No labeling was observed under this condition.

### 2.5 Parasite Adhesion Assays

Alcian blue and fibronectin were used in promoting cell adhesion to glass coverslips. Alcian blue-coated coverslips were prepared as previously described (Morone et al., 2006). Fibronectin-coated coverslips were prepared by first covering them with 100 µl of human (Sigma F0556) or bovine (Sigma F01141) fibronectin (working solution of 10 µg/ml in sterile PBS) for 1 h at room temperature and washing them with sterile PBS. Parasites (1 × 10^6^ cells/ml) were washed in PBS (pH 7.2) and resuspended in TYM medium without serum and PBS for Alcian blue and fibronectin assays, respectively. A suspension of 50 µl was incubated on 1% Alcian blue or fibronectin-coated glass coverslips in a humidity chamber for 0.5 to 2 h at 37°C. The parasite adhesion was monitored using an inverted phase-contrast microscope. Non-adherent cells were collected with a pipette, harvested by centrifugation, and washed with PBS. Next, the coverslips were rigorously washed with PBS to remove non-adherent parasites. Adherent cells remain on the coverslips even after several washes. Both adherent and non-adherent cells were then fixed and analyzed using scanning electron microscopy (SEM) as mentioned above. For the control experiments, parasites resuspended in TYM medium without serum or PBS were incubated on uncovered coverslips under the same conditions, collected with a pipette, harvested by centrifugation, and analyzed as mentioned above.

### 2.6 Parasite–Host Cell Interaction

The human HeLa cells (ATCC CCL-2) were grown in DMEM complemented with 10% bovine fetal serum, 10 U/ml penicillin, and 10 μg/ml streptomycin (Invitrogen) and cultured at 37°C/5% CO_2_. HeLa cells were seeded onto 24-well tissue culture plates in DMEM medium and allowed to form a confluent monolayer (1 × 10^6^ cells) at 37°C in 5% CO_2_. Fresh bovine preputial epithelial cells (PECs) were kindly provided by Dr. Maria Aparecida da Gloria Faustino from the Faculty of Veterinary Medicine/Rural Federal University of Pernambuco. PECs were collected by aspiration with an artificial insemination pipette or by scraping the preputial cavity from a mature bull (>4 years old) and suspended in 50 ml of warm (37°C) PBS (pH 7.2) just prior to the experiments. Next, HeLa and PECs were washed two times in warm PBS by centrifugation at 400×*g* for 5 min, suspended to a cellular density of 10^5^ cells/ml in warm PBS, and immediately used for interaction assays. HeLa and PECs were co-incubated with *T. vaginalis* and *T. foetus*, respectively, at cell ratios of 1:1 or 5:1 parasite:host cell in PBS-F [PBS with 1% fetal bovine serum (FBS) at pH 6.5] at 37°C for 30 min. Prior to the co-incubation, parasites were washed three times in PBS, pH 7.2, and incubated to PBS-F at 37°C for 15 min. In some assays, the human benign prostate epithelial line BPH1 was grown as described (Twu et al., 2013) and co-incubated with *T. vaginalis* as described above. For the control experiments, parasites incubated in PBS in the absence of host cells were analyzed. The interactions were analyzed using SEM, as mentioned above.

### 2.7 Immunofluorescence Assays

Parasites expressing the hemagglutinin tag (HA) version of TvVPS32 and TfVPS32 were incubated at 37°C on glass coverslips for 4 h as previously described (Coceres et al., 2015). The parasites were then fixed and permeabilized in cold methanol for 10 min. Cells were then washed and blocked with 5% FBS in PBS for 30 min, incubated with a 1:500 dilution of anti-HA primary antibody (Covance, Emeryville, CA, USA) and 1:500 dilution of anti-tubulin primary antibody diluted in PBS plus 2% FBS for 2 h at RT, washed with PBS, and then incubated with a 1:5,000 dilution of Alexa Fluor-conjugated secondary antibody (Molecular Probes) 1 h at RT. The coverslips were mounted onto microscope slips using ProLong Gold antifade reagent with 4,6′-diamidino-2-phenylindole (Invitrogen). All observations were performed on a Nikon E600 epifluorescence microscope. Adobe Photoshop (Adobe Systems) was used for image processing.

### 2.8 Motility Assay

Parasites TvEpNeo and TvVPS32 (1 × 10^6^ cells) were inoculated in soft-agar plates with Diamond’s, 5% FBS, 0.32% agar, and 10 U/ml penicillin/10 μg/ml streptomycin (Invitrogen). Parasite migration was monitored by analyzing the colony diameter during 4 days under microaerophilic conditions at 37°C. Halo diameter was determined by ImageJ (image processing program).

### 2.9 Quantitative Analysis

The measurement of EAS filaments was carried out using TEM Imaging & Analysis (TIA) software of the microscope (FEI Company). The percentage of parasites that contain flagellar swelling was determined from counts of at least 500 parasites randomly selected per sample, using SEM or light microscope. The quantification of morphological aspects and the distribution of flagellar swellings per cell were determined from counts of 100 parasites displaying at least one swelling per sample, using SEM. The morphology and relative position of flagellar swelling per flagellum was determined from counts of at least 100 anterior and recurrent flagella with swelling per sample, using SEM. The number of rosettes/µm^2^ was determined from counts of 50 flagella with or without swellings from at least 10 random fields in the transmission electron microscopy (TEM) grids using the TIA software. The results are the average of three independent experiments performed at least in duplicate. Statistical comparison was performed (ANOVA test), using computer analysis (GraphPad Prism v. 7.04, CA, USA). *p <*0.05 was statistically significant.

## 3 Results

### 3.1 Presence of Flagellar Swellings in *Trichomonas vaginalis* and *Tritrichomonas foetus*

**Figure 1:**
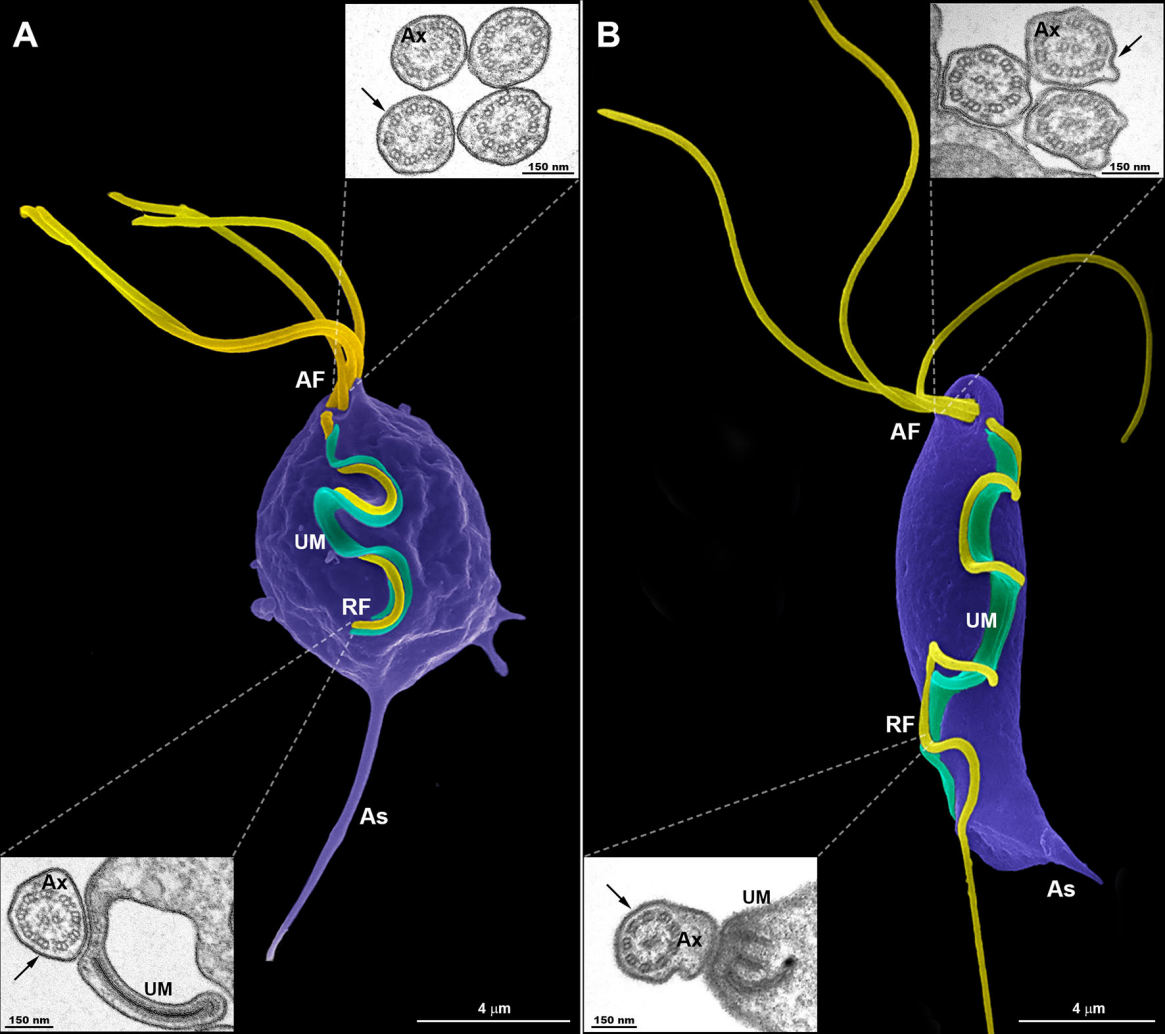
Typical morphology of trichomonads grown in axenic culture. SEM of *Trichomonas vaginalis* **(A)** and *Tritrichomonas foetus* **(B)** with the pear-shaped cell bodies colored violet and the flagella colored yellow. *Trichomonas vaginalis* exhibits four anterior flagella (AF), whereas *T. foetus* has three AF; both parasites have one recurrent flagellum (RF) that runs posteriorly along the cell body, forming an undulating membrane (UM—colored green). The *T. vaginalis* RF is shorter than the *T. foetus* RF. The latter displays a distal free end. The flagella are the same width along their length and no swellings or enlarged areas are seen. The axostyle (As) tip is visible. The insets are TEM images of the AF (upper insets) and RF (lower insets) in representative transverse sections, viewed from the proximal and distal end, respectively. Note the 9 + 2 axoneme (Ax) enclosed within the flagellar membrane (arrows). No extra-axonemal structures are seen.

**Figure 2:**
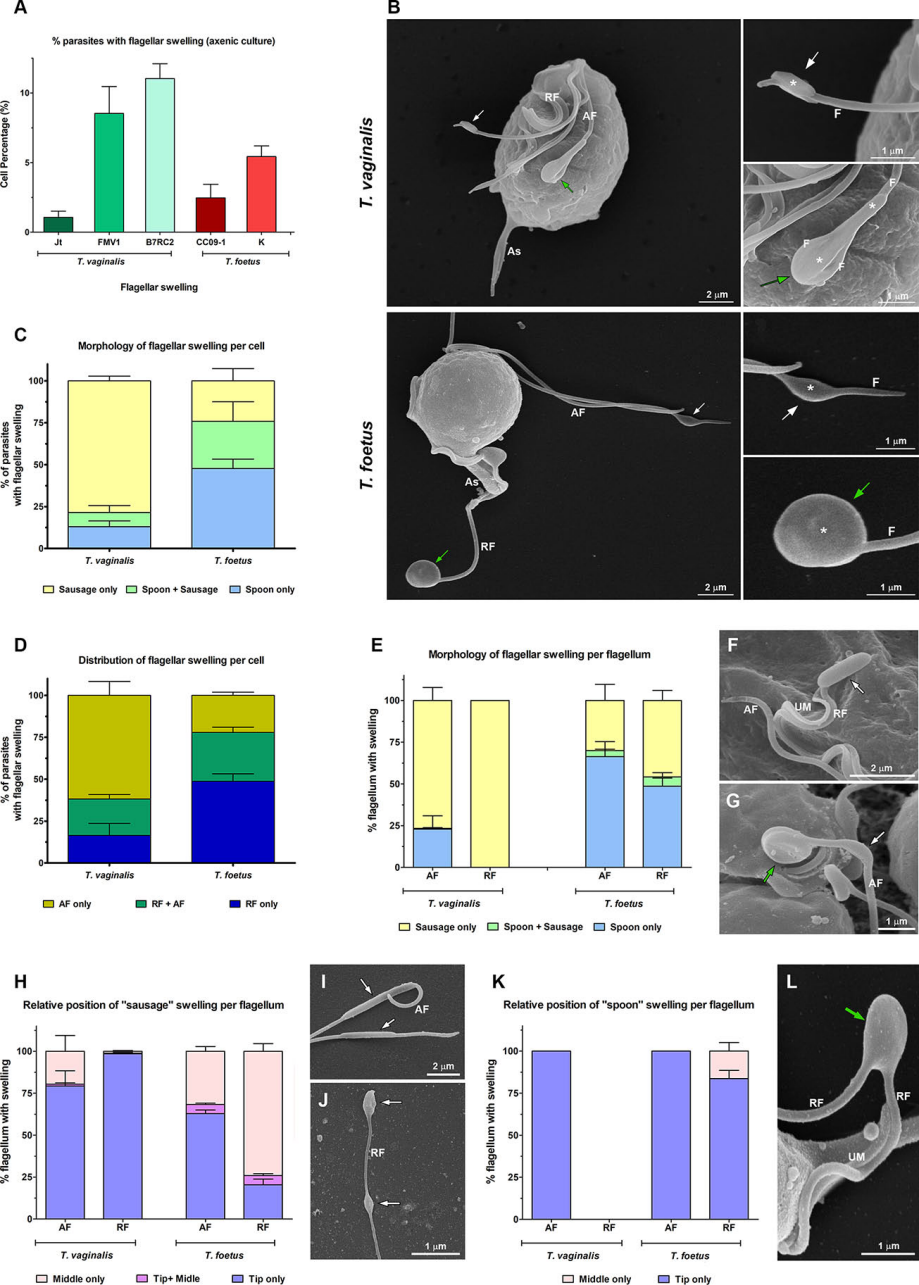
Morphological analyses of flagellar swellings in *Trichomonas vaginalis* and *Tritrichomonas foetus* under standard growth conditions. **(A)** Quantification of the percentage of parasites that display flagellar swellings. The values are expressed as the means ± standard deviation (SD) of three independent experiments, each performed in duplicate. Five hundred parasites per sample were randomly counted. **(B)** General and detailed views of flagellar swellings (*) in *T. vaginalis* and *T. foetus* obtained by SEM. The swellings can exhibit two different morphologies: “sausage-shaped” (white arrows) and “spoon-shaped” (green arrows). Notice that the “sausage-like” swelling runs laterally to the flagellum **(F)**, whereas in the spoon-shaped structure, the swelling is surrounded by the flagellum. AF, anterior flagella; RF, recurrent flagellum; As, axostyle. **(C, D)** Quantitative analysis of the morphology **(C)** and distribution **(D)** of flagellar swellings per parasite. Three independent experiments in duplicate were performed, and 100 parasites exhibiting at least one swelling were randomly counted per sample using SEM. Data are expressed as percentage of parasites with flagellar swelling ± SD. AF, anterior flagella; RF, recurrent flagellum. **(E)** Quantification of the morphology of flagellar swelling per flagellum. The values are expressed as the means of the percentage of flagellum with swelling ± SD of three independent experiments, each performed in duplicate. One hundred anterior and recurrent flagella with swelling per sample were randomly counted using SEM. AF, anterior flagella; RF, recurrent flagellum. **(F, G)** Detailed views of RF of *T. vaginalis* **(F)** and AF of *T. foetus* **(G)** by SEM. UM, undulating membrane. In **(F)**, a sausage-shaped swelling (arrow) is seen at the tip of the flagellum. Notice in **(G)** the presence of “sausage” (white arrow) and “spoon-like” (green arrow) structures in the same flagellum. **(H)** Analysis of the relative position of “sausage” swelling per flagellum. Three independent experiments in duplicate were performed, and 100 anterior and recurrent flagella with swelling per sample were randomly counted using SEM. Data are expressed as percentage of flagellum exhibiting swelling ± SD. AF, anterior flagella; RF, recurrent flagellum. **(I, J)** SEM of sausage-shaped structures (arrows) located along the AF of *T. vaginalis* **(I)** and at the tip and in the middle of the same recurrent flagellum of *T. foetus* **(J)**. **(K)** Quantification of the relative position of “spoon” swelling per flagellum. The values are expressed as the means of the percentage of flagellum exhibiting swelling ± SD of three independent experiments, each performed in duplicate. One hundred anterior and recurrent flagella with swelling per sample were randomly counted using SEM. AF, anterior flagella; RF, recurrent flagellum. **(L)** SEM of a spoon-shaped structure (arrow) located in the middle of *T. foetus* RF. UM, undulating membrane.

To examine in detail the trichomonad flagellar morphology, we initially observed three wild-type strains of *T. vaginalis* and two different strains of *T. foetus* grown axenically using SEM and TEM. As can be visualized in **Figure 1**, *T. vaginalis* has four anterior flagella (AF), *T. foetus* has three AF, and both parasites have one recurrent flagellum (RF) that forms the undulating membrane. In *T. vaginalis*, the RF runs along two-thirds of the cell and no free portion is developed, whereas in *T. foetus*, the RF reaches the posterior end of the cell and extends beyond the undulating membrane as a free tip (**Figure 1**). As expected, the flagella of most of the parasites (between 89% and 99%) displayed a classical ultrastructure: a diameter of 250–300 nm along their length and the flagellar membrane around the “9 + 2” axoneme (**Figure 1**, insets). However, the presence of flagellar swellings in the tip or along the AF and RF was observed in 1%–11% of *T. vaginalis* and 2%–5% of *T. foetus* parasites analyzed by SEM (**Figure 2A**). The flagellar swellings were initially analyzed in three strains for *T. vaginalis* (Jt, FMV1, B7RC2) and two strains for *T. foetus* (CC09-1 and K), and the strains B7RC2 and K were subsequently used. These swellings exhibited two different morphologies: “sausage-like” and “spoon-like” (**Figure 2B**). The “sausage-like” swelling runs laterally or surrounding the axoneme, exhibiting a range size from 0.1 to 1 µm in thickness and a variable length from 0.3 to 6 µm in *T. vaginalis* and up to 1 µm in *T. foetus* (**Figure 2B** and **Supplementary Figure 1**). In the “spoon-like” swelling, the flagellum wraps around the swelling to form a rounded or ellipsoid structure measuring between 0.5 and 2.5 µm in the major axis in *T. foetus* and more than 4 µm long in *T. vaginalis* (**Figure 2B** and **Supplementary Figures 2A–C**). The “spoon-like” structure can exhibit a flattened or concave surface in frontal view (**Supplementary Figures 2D, E**) and an aligned, curved, or convex appearance on side view (**Supplementary Figures 2F–H**). Curiously, while the “sausage-like” structure was more frequently found in *T. vaginalis*, the “spoon-like” was more common in *T. foetus* (**Figure 2C**). Interestingly, although these structures can be found in all flagella, they are more frequent in the AF in *T. vaginalis* and RF in *T. foetus* (**Figure 2D**). The analysis of “spoon-like” and “sausage-like” flagellar distribution demonstrates that both types of structures can be identified in the RF and AF in *T. foetus* as well as in the AF of *T. vaginalis* (**Figure 2E**). However, only “sausage-like” structures were detected in the RF of *T. vaginalis* (**Figures 2E, F**). In *T. foetus*, around 3%–6% of flagella with swelling exhibited “sausage-like” and “spoon-like” structures in the same flagellum (**Figures 2E–G**). When the relative position of both types of structures along the flagella was evaluated, we noted that the “sausage-like” swelling was predominantly found at the flagellar tip of *T. vaginalis* and AF of *T. foetus* (**Figure 2H**); however, it was also observed in the middle (**Figures 2H, I**) and, rarely, at the tip and in the middle of the same flagellum (**Figures 2H–J**). The “spoon-like” structure was usually located at the tip of AF of both parasites and, occasionally, seen in the middle of *T. foetus* RF (**Figures 2K, L**).

### 3.2 Flagellar Swellings Are Extra-Axonemal Structures Formed by Thin Filaments

**Figure 3:**
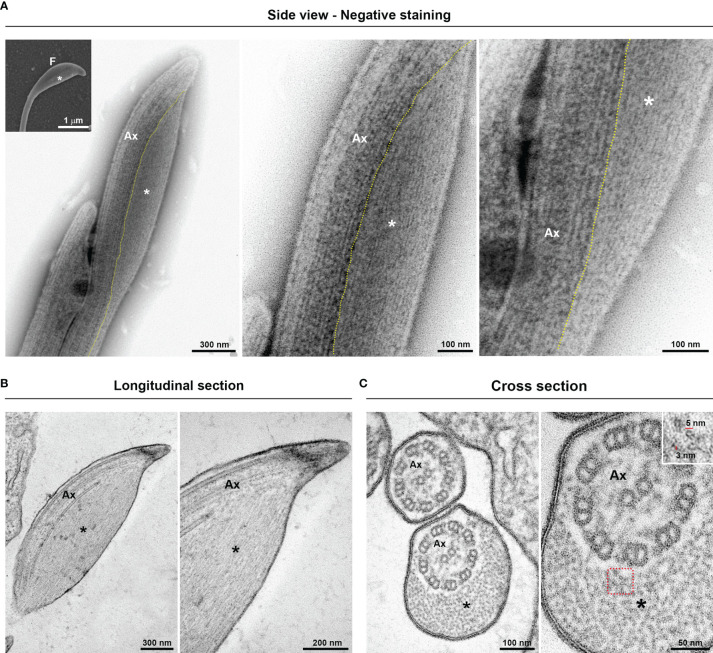
Ultrastructure of the flagellar sausage-shaped swelling. The structure is formed by thin extra-axonemal filaments (*) that run longitudinally along the axoneme (Ax). **(A)** Negative staining images of a swelling on side view. The dotted lines indicate the boundary between axoneme and the extra-axonemal filaments. Inset, a complementary SEM image is used as reference. F, flagellum. **(B, C)** Longitudinal and cross ultrathin sections. The extra-axonemal filaments measure around 3–5 nm in diameter (inset).

**Figure 4:**
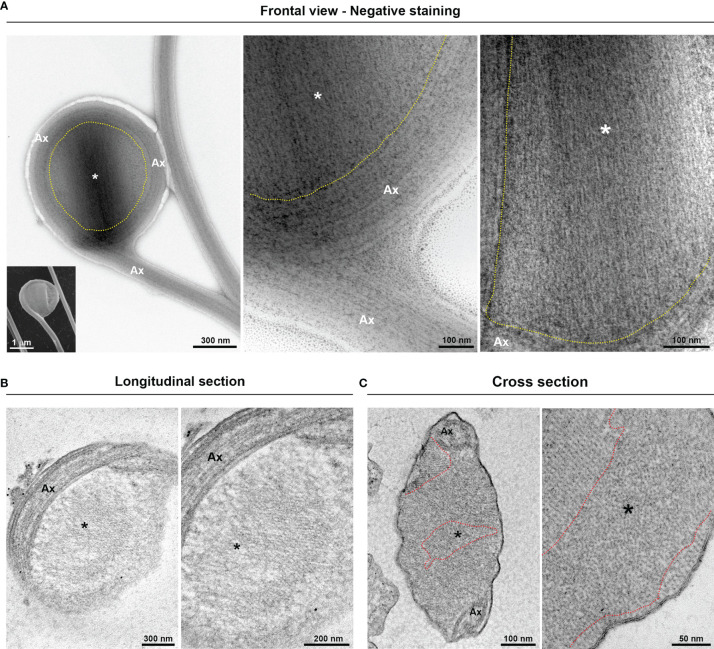
Fine structure of the flagellar “spoon-like” swelling. The structure is formed by folding the axoneme (Ax) around the thin extra-axonemal filaments (*). **(A)** Negative staining images of a swelling on frontal view. The dotted lines indicate the boundary between axoneme and the extra-axonemal filaments. Inset, a complementary SEM image is used as reference. **(B)** Longitudinal ultrathin sections. The extra-axonemal filaments display a lattice-like arrangement. **(C)** Cross ultrathin sections. The filaments are seen organized in different orientations, as indicated by the dotted lines.

To investigate the ultrastructural characteristics of flagellar swellings in trichomonads, we analyzed the flagella using negative staining and ultrathin section techniques for TEM (**Figures 3**, **4**). Our results demonstrate that flagellar microtubules are surrounded by a continuous membrane that comes from the cell body and that a “sausage-like” swelling is formed by thin extra-axonemal filaments that run longitudinally along the axonemes (**Figure 3**). A detailed analysis of longitudinal and transverse sections showed that the extra-axonemal filaments measure around 3–5 nm in diameter and their length varies according to the length of the swelling (**Figure 3C**). To further understand the morphological organization of “sausage” swelling, we analyzed complementary images acquired in different perspectives (**Supplementary Figure 3**). Those results confirmed that the extra-axonemal filaments partially surround the axoneme, although SEM top view images may lead to misinterpretation of the flagella being totally surrounded by the swelling (**Supplementary Figure 3**). In an oblique view, we noticed that the axoneme is in a slit of the swelling as a hot dog-shaped structure (**Supplementary Figure 3**). The “sausage” structures located in the middle of the flagella and in the RF are also formed by extra-axonemal filaments (**Supplementary Figures 4**, **5**), indicating that the flagellar swellings are EASs.

**Figure 5:**
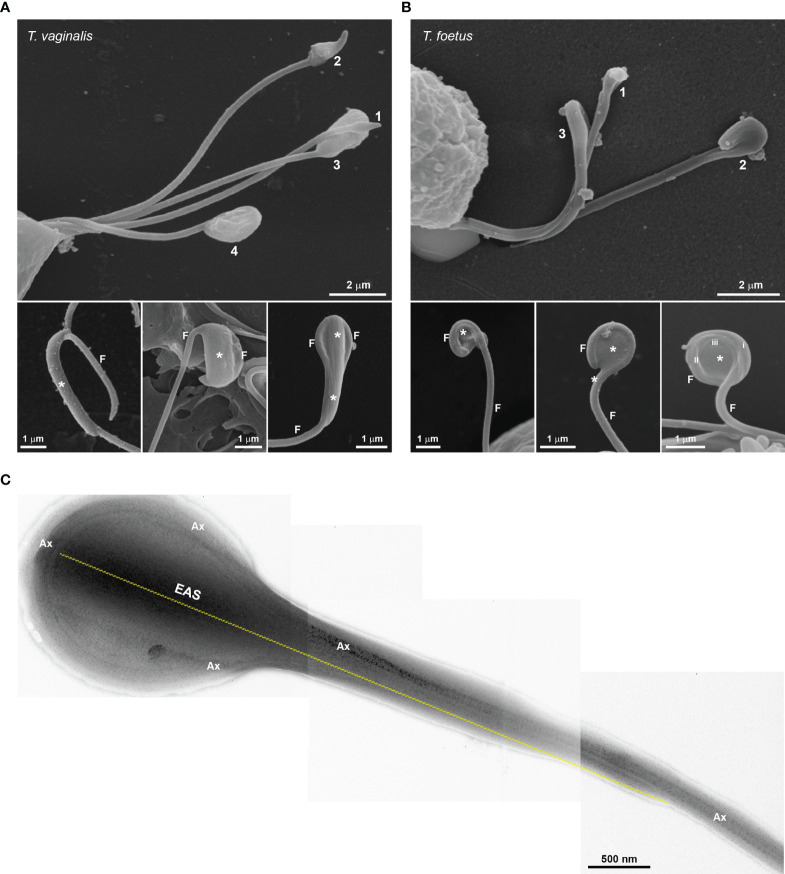
Morphological diversity of flagellar swelling. **(A, B)** SEM showing swellings (*) of different sizes in *Trichomonas vaginalis* **(A)** and *Tritrichomonas foetus* **(B)**. Numbers 1, 2, 3, and 4 and lower images suggest plausible stages for the spoon-shaped structure formation. In **(B)**, the roman numbers (i, ii, and iii) indicate the amount of flagellum (F) folds around the swelling. **(C)** Negative staining of a sausage-shaped extra-axonemal structure (EAS) surrounded by axoneme (Ax). Dotted line indicates the EAS length.

Additionally, we demonstrated that the “spoon-type” swelling is also an EAS formed by folding the axoneme around the extra-axonemal filaments (**Figure 4A**). When observed in longitudinal sections, the filaments display a lattice-like arrangement (**Figure 4B**). In a transversal view, it can be observed that the filaments are organized in different orientations (**Figure 4C**), probably due to the turns of the axoneme around the filaments. As swellings are formed by extra-axonemal filaments, it is very likely that morphological differences could be attributed to different phases of a single process. Supporting this, SEM analysis suggests that the “sausage” and the “spoon” could be different stages of a single event (**Figures 5A, B**). The process might start with a small sausage-shaped EAS that gives rise to a “spoon” when the flagella fold around an enlarged EAS and on themselves (**Figures 5A, B**). TEM images confirmed that a sausage-shaped EAS is surrounded by axoneme (**Figure 5C**). Because the *T. vaginalis* RF has no free portion, this could help explain why only sausage-shaped EASs are observed in that flagellum, whereas both sausage- and spoon-shaped EASs are found in the free tip of *T. foetus* RF (**Supplementary Figure 6**).

**Figure 6:**
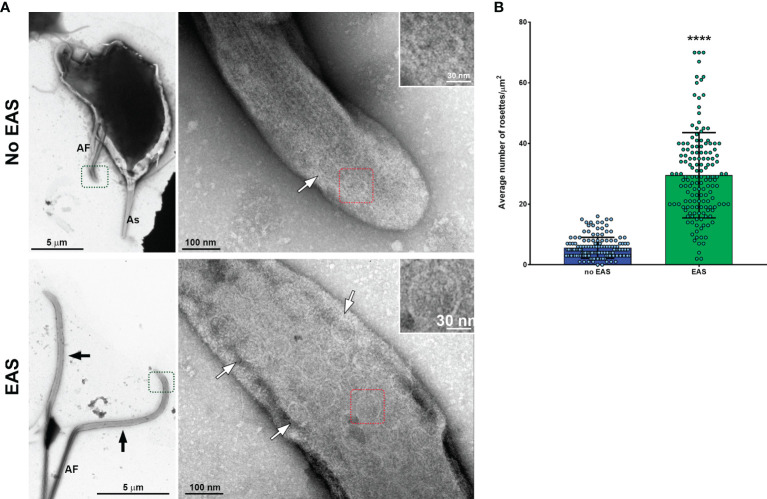
Flagella with swelling exhibit a higher number of rosette-like formations. **(A)** Representative general and detailed views of *Trichomonas vaginalis* anterior flagella (AF) without and with extra-axonemal structures (EAS) obtained by TEM. Many rosette-like formations (white arrows) are seen in the flagella with swelling (black arrows). As, axostyle. **(B)** Quantification of the number of rosettes/µm^2^. The columns represent the average number of rosettes/µm^2^ ± standard deviation (SD) of three independent experiments. Fifty flagella with or without swellings per sample were randomly counted using TEM. The dots indicate the values obtained for each flagellum. Flagella with EAS show a higher number of rosettes/µm^2^ than those flagella without EAS. ****p < 0.0001 compared with the “no-EAS” group using non-parametric *t*-test (Mann–Whitney test).

The existence of rosette-like formations (clusters of intramembrane particles), proposed as sensorial structures, has been reported in the AF of *T. vaginalis* and *T. foetus* (Benchimol et al., 1981; Benchimol and De Souza, 1990). In this regard, we evaluated the presence of rosettes in the *T. vaginalis* EASs by negative staining technique. Interestingly, we observed that flagella with EASs showed a higher number of rosettes/µm^2^ than those flagella without such structures (**Figure 6**). In summary, our results demonstrated that EASs in trichomonads are membrane expansions with different morphologies (sausage/spoon), formed by thin filaments and a high number of rosettes in their membranes.

### 3.3 The EAS Formation Increases During *Trichomonas vaginalis* and *Tritrichomonas foetus* Attachment Process

**Figure 7:**
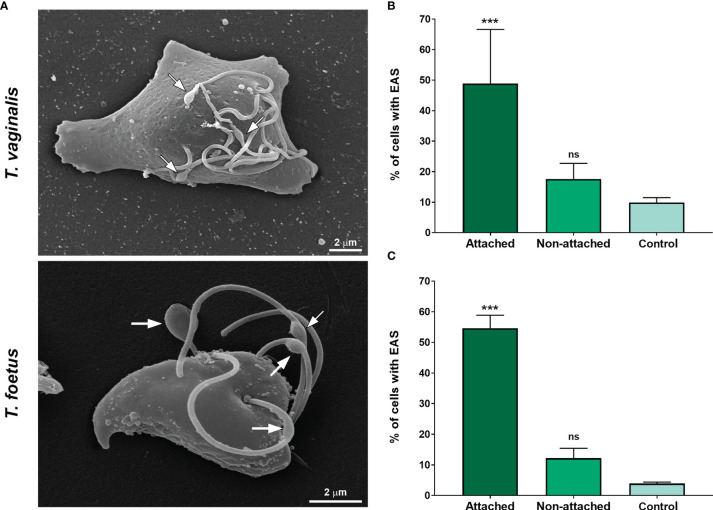
The EAS formation increases during trichomonad attachment on fibronectin-coated coverslips. **(A)** SEM of *Trichomonas vaginalis* and *Tritrichomonas foetus* after adhesion assay on fibronectin-coated coverslips. Arrows indicate the EAS. Notice that parasites display an amoeboid morphology. **(B, C)** Quantitative analyses in *T. vaginalis* **(B)** and *T. foetus* **(C)**. The percentage of cells with EASs was determined by counting 500 parasites per sample using SEM. Data are expressed as means of three independent experiments in duplicate ± SD. Attached and non-attached: parasites resuspended in PBS incubated on fibronectin-coated coverslips in a humidity chamber for 2 h at 37°C and rigorously washed with PBS to remove non-attached cells. Attached parasites remain on the coverslips even after several washes. Non-attached parasites were collected with a pipette, harvested by centrifugation, and prepared for SEM. Control, parasites incubated on uncoated coverslips under the same conditions mentioned above, collected with a pipette, harvested by centrifugation, and prepared for SEM. “Control” is formed by non-adherent, suspended cells from uncovered coverslips, whereas non-adherent parasites from fibronectin are called “non-attached.” The percentage of parasites displaying EASs is significantly higher in the attached group when compared with the non-attached and control groups. ***p < 0.001 compared with the control group using one-way ANOVA test (Kruskal–Wallis test; Dunn’s multiple comparisons test). ns, non-significant.

The ability of trichomonads to colonize the epithelia has been studied in recent years; however, the role of flagella in this process is not fully understood. To evaluate a possible correlation of extra-axonemal structures to parasite attachment, parasites were incubated on fibronectin-coated coverslips or Alcian blue precationized coverslips, washed with PBS to remove non-attached cells, and the formation of EASs was evaluated by SEM (**Figure 7** and **Supplementary Figure 7**). The attached parasites remained on the coverslips, whereas non-attached cells were harvested by centrifugation and also analyzed. For control, parasites were incubated on uncoated coverslips, collected with a pipette, harvested by centrifugation, and also prepared for SEM. As expected, cells were in suspension and unattached on the uncoated coverslips (not shown); therefore, here, “control” was defined as non-adherent, suspended cells from uncoated coverslips, whereas non-adherent parasites from fibronectin and Alcian blue interaction assays were called “non-attached.” Parasites from control exhibited the typical pyriform body and no cell clusters (**Supplementary Figure 7**). As expected, the attached parasites on fibronectin-coated coverslips exhibited an amoeboid morphology and many flagellar swellings (**Figure 7A**). The percentage of fibronectin-attached parasites with EAS was higher when compared with the non-attached and control groups (**Figures 7B, C**). In control, EASs were found in 9.9% and 3.9% of *T. vaginalis* and *T. foetus*, respectively, whereas EAS formation was observed in 48.9% and 54.6% of fibronectin-attached *T. vaginalis* and *T. foetus* groups, respectively (**Figures 7B, C**). When the parasites were incubated onto coverslips pretreated with Alcian blue, the cells were found clustered, mainly *T. vaginalis*, displaying an amoeboid or ellipsoid form in both attached and non-attached groups (**Supplementary Figure 7A**). Similarly, the percentage of parasites with EAS in the Alcian blue-attached parasite was higher when compared with control (**Supplementary Figures 7B, C**). In control, EASs were found in 12.5% and 5.2% of *T. vaginalis* and *T. foetus*, respectively, whereas EAS formation was observed in 41.7% and 40.2% of Alcian blue-attached *T. vaginalis* and *T. foetus* groups, respectively (**Supplementary Figures 7B, C**). Unexpectedly, the percentage of *T. vaginalis* with EAS in the Alcian blue non-attached group was significantly higher when compared with control (**Supplementary Figures 7B, C**).

**Figure 8:**
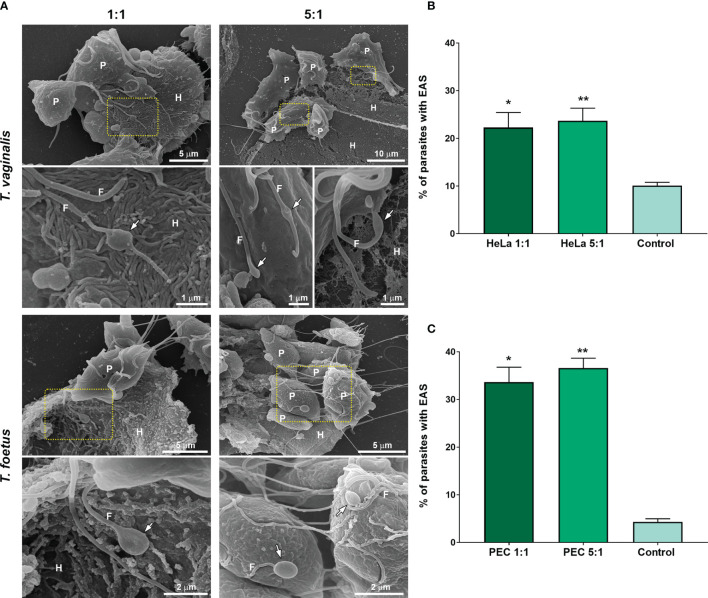
EASs are formed in response to host cell exposure. **(A)** Representative SEM images of *Trichomonas vaginalis* and *Tritrichomonas foetus* after host cell interaction. HeLa and bovine preputial epithelial cells (PECs) were co-incubated with *T. vaginalis* and *T. foetus*, respectively, at cell ratios of 1:1 or 5:1 parasite:host cell in PBS-F (PBS with 1% FBS at pH 6.5) at 37°C for 30 min. Flagellar swelling (arrows) are seen in some parasites (P). Notice that some swellings are in direct contact to the host cells (H). **(B, C)** Quantification of the percentage of *T. vaginalis* **(B)** and *T. foetus* **(C)** with flagellar swelling after the host cell interaction. Three independent experiments in duplicate were performed, and 500 parasites were randomly counted per sample using SEM. Data are expressed as percentage of parasites ± SD. For the control experiments, parasites incubated in PBS in the absence of host cells were analyzed. The percentage of parasites with flagellar swelling increases after the hot cell exposure when compared with control (PBS). *p < 0.05; **p < 0.01 compared with control using one-way ANOVA test (Kruskal–Wallis test; Dunn’s multiple comparisons test).

Next, to evaluate if EASs could have a role in epithelial cells interaction, parasites were incubated with target cells and the number of parasites with flagellar swellings was quantified using SEM (**Figure 8**). Two different ratios of parasites:host cells were used, and parasites in the absence of target cells were used as control (PBS). Upon exposure, EASs of different sizes were found in some parasites and some swellings were seen in direct contact with the host cells (**Figure 8A** and **Supplementary Figure 8**). When *T. vaginalis* parasites were incubated with HeLa at 1:1 and 5:1 ratios, the formation of EASs was observed in 22.3% and 23.7% of the parasites, respectively (**Figure 8B**). Similarly, when *T. foetus* were exposed to PECs (bovine preputial epithelial cells), EASs were observed in 33.6% and 36.5% of the attached parasites at ratios of 1:1 and 5:1, respectively (**Figure 8C**). Moreover, we observed that these structures were present in the flagella of parasites in contact with prostatic cells, preputial mucus, and bacteria present in the microbiota of the reproductive system (**Supplementary Figure 9**). Together, these results suggest that the formation of extra-axonemal structures increases in response to host cell exposure.

### 3.4 Microvesicle-Like Structures Are Shed From the Membrane of EASs

**Figure 9:**
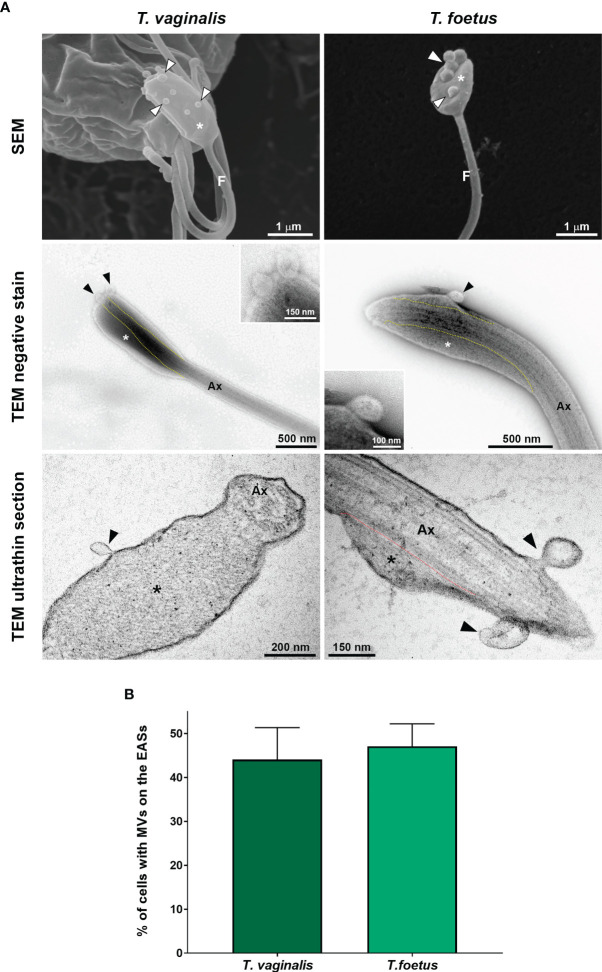
EASs release microvesicle-like structures. **(A)** Representative micrographs of MVs (arrowheads) protruding from the flagellar membrane of the EASs (*) of *Trichomonas vaginalis* and *Tritrichomonas foetus*. The images were obtained by SEM (first row), negative staining (second row), and ultrathin sections (third row). The dotted lines indicate the boundary between axoneme (Ax) and the extra-axonemal filaments (*). **(B)** Percentage of EASs with protruding MVs on their surface. Three independent experiments in duplicate were performed, and 100 parasites exhibiting at least one swelling were randomly counted per sample using SEM. Data are expressed as means ± SD. Approximately 45% of parasites with flagellar swelling exhibited associated MVs.

Flagella can send information through microvesicles (MVs) released from their membranes (Wood et al., 2013; Szempruch et al., 2016). Previous results from our group demonstrated that *T. vaginalis* releases flagellar MV-like structures, although their biological relevance is still unknown (Nievas et al., 2018). Here, we observed the presence of MV-like structures associated to EASs by SEM, negative staining, and ultrathin sections (**Figure 9A**). In axenic culture, we demonstrated that 44.1% and 47.1% of *T. vaginalis* and *T. foetus* with flagellar swelling, respectively, exhibit MVs protruding from the flagellar membrane of the EASs (**Figure 9B**). Considering the presence of MV-like structures in the EAS membrane and the role of MVs in intercellular communication, these results suggest a possible role of MVs protruding from EASs in cell-to-cell communication.

### 3.5 VPS32 Localizes to the EASs and Its Overexpression Increases EAS Formation in *Trichomonas vaginalis* and *Tritrichomonas foetus*

**Figure 10:**
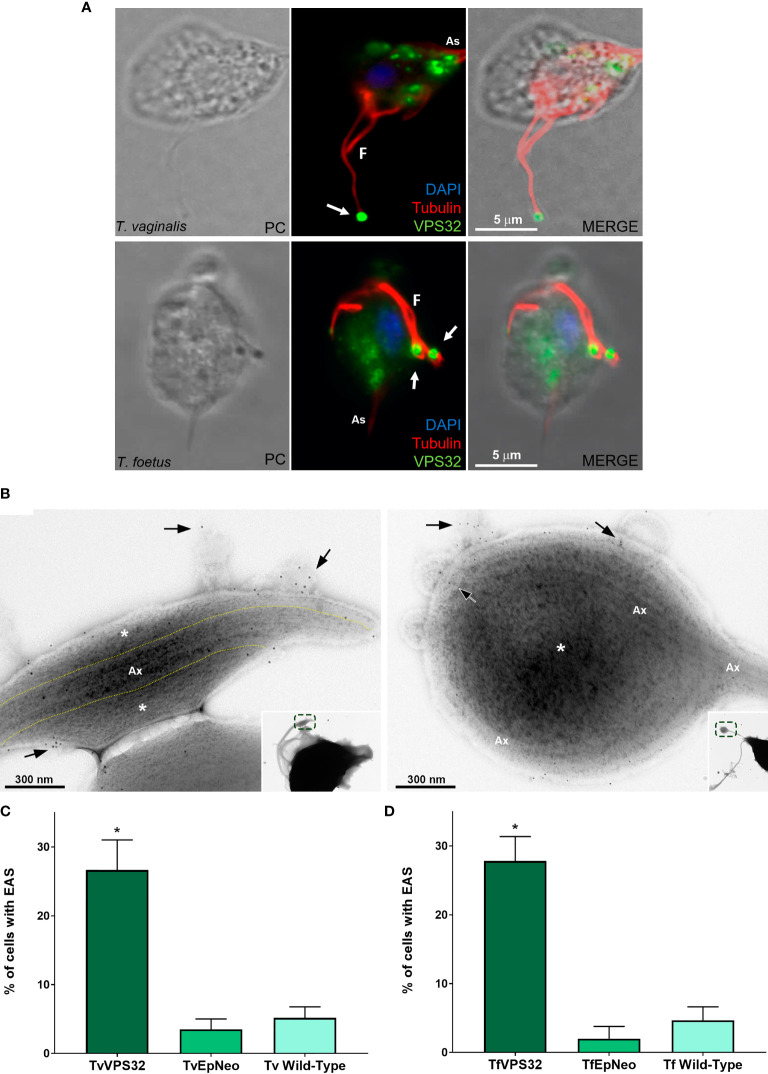
VPS32 is present in EAS surface, and its overexpression increases EAS formation. **(A)** Representative immunofluorescence microscopy images of *Trichomonas vaginalis* and *Tritrichomonas foetus* exogenously expressing TvVPS32 and TfVPS32 with a C-terminal hemagglutinin (HA) tag, respectively, using a rabbit anti-HA antibody (green). PC, phase-contrast image. The flagella (F) and axostyle (As) are labeled with mouse anti-tubulin antibody (red). Arrows indicate the subcellular localization of VPS32 in structures similar to flagellar swelling at flagella tip. The nucleus (blue) is stained with 4′,6′-diamidino-2-phenylindole (DAPI). The cytosolic subcellular localization of VPS32 protein is also noticed. **(B)** Negative staining of TvVPS32-HA-transfected parasites immunogold-labeled with anti-HA antibody demonstrates that TvVPS32 is localized in the surface of extra-axonemal structures (EASs) as well as in MVs that protrude from EASs (arrows). **(C–D)** Analysis of the percentage of EASs in the flagella of TvVPS32FL **(C)** and TfVPA32FL **(D)** parasites. Three independent experiments in duplicate were performed, and 100 parasites exhibiting at least one swelling were randomly counted per sample using a phase-contrast microscope. Data are expressed as means ± SD. Approximately 27% and 28% of flagellar EASs were observed in TvVPS32- and TfVPS32-transfected parasites, respectively, compared with 2%–5% of EASs observed in EpNeo (empty plasmid transfected) and wild-type parasites. *p < 0.05 compared with EpNeo and wild-type parasites using one-way ANOVA test (Kruskal–Wallis test; Dunn’s multiple comparisons test).

The ESCRT-III complex is a key player in the regulation of membrane fission during MV formation and membrane remodeling (McCullough et al., 2018). VPS32 is an important component of the ESCRT-III complex (Cashikar et al., 2014). Hence, we transfected an HA-tagged version of the full-length protein (VPS32FL-HA) in *T. vaginalis* (TvVPS32) and *T. foetus* (TfVPS32) to evaluate its localization by epifluorescence microscopy. As expected, TvVPS32 and TfVPS32 were observed in cytosolic vesicles (**Figure 10A**), as previously reported by our group (Iriarte et al., 2018). In addition to this cytosolic localization, we demonstrated that VPS32 protein is also located in structures similar to the “spoon” swelling at the flagellar tip of parasites cultured in the absence of host cells (**Figure 10A**). In concordance, the presence of VPS32 in the EAS surface, as well as in MVs, that protrudes from EASs was observed by immuno-gold electron microscopy using anti-HA antibody (**Figure 10B** and **Supplementary Figure 10**). Importantly, few or no gold particles were found in the regions without EASs (**Supplementary Figure 10**), confirming the specificity of immunolabeling. Based on this observation, we investigated the correlation between VPS32 and EAS formation by analyzing the number of EASs in the flagella of TvVPS32FL and TfVPA32FL parasites compared with parasites transfected with an empty plasmid (EpNeo). Interestingly, 27% and 28% of TvVPS32- and TfVPS32-transfected parasites exhibited EAS, respectively, compared with 2%–5% EpNeo and wild-type parasites (**Figures 10C, D**). We also observed MV-like structures protruding from EASs of transfected cells (**Supplementary Figure 11A**). The percentage of VPS32-overexpressing parasites with MVs on the EASs was 1.6-fold higher when compared with control cells (**Supplementary Figure 11B**). Moreover, we demonstrated that parasites overexpressing VPS32 have a striking increase in adherence to fibronectin-coated coverslips, approximately 2.4-fold, compared with control parasites (**Supplementary Figure 11C**). Importantly, the VPS32 expression in the transfected parasites was confirmed by Western blot using an anti-HA antibody (**Supplementary Figure 11D**).

### 3.6 TvVPS32 Might Regulate Parasite Motility

**Figure 11:**
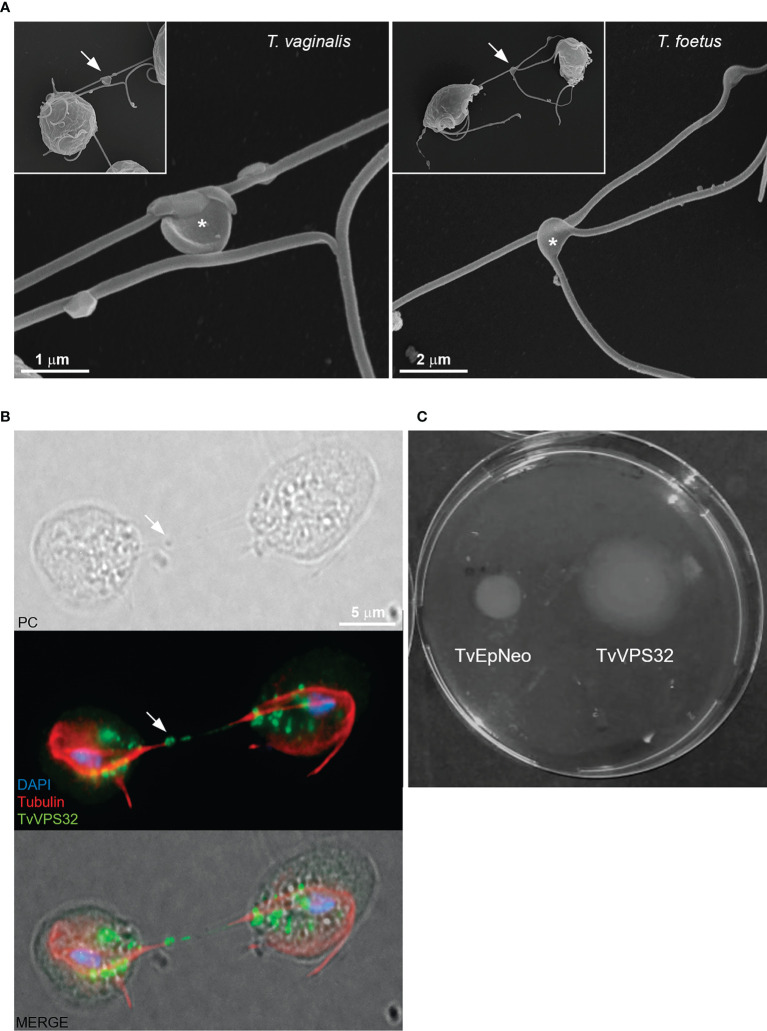
TvVPS32 might play a role in parasite motility. **(A)** Representative SEM images of parasites (*Trichomonas vaginalis* and *Tritrichomonas foetus*) connected to themselves by EASs (arrows). Notice the EASs in higher magnification (*). **(B)** Immunofluorescence images showing that TvVPS32-transfected parasites connect with each other through the flagella and that TvVPS32 is localized in the flagella of parasites in contact. TvVPS32 parasites cultured in the absence of host cells were co-stained with anti-HA (green) and tubulin (red). The nucleus (blue) was also stained with DAPI. Arrows indicate the EASs. PC, phase-contrast image. The cytosolic subcellular localization of VPS32 protein is also noticed. **(C)** Representative TvVPS32 parasite motility assay. TvEpNeo (empty plasmid transfected) and TvVPS32 parasites were spotted onto soft agar, and their migration capacity was analyzed by measuring the size of the halo diameter during 4 days under microaerophilic conditions at 37°C. TvVPS32 parasites showed a higher capacity of migration compared with TvEpNeo parasites.

Information exchange between parasites of the same species could govern the decision to divide, to differentiate, or to migrate as a group (Roditi, 2016). In some cases, this communication involves flagellar membrane fusion and the rapid exchange of proteins between connected cells (Szempruch et al., 2016). In this sense, our SEM observations demonstrate that *T. vaginalis* and *T. foetus* can connect to themselves by EASs present in flagella (**Figure 11A**). Similarly, we observed that TvVPS32-transfected parasites can connect each other through the flagella and that TvVPS32 is localized in the flagella of parasites in contact (**Figure 11B**). Based on this observation, we next decided to indirectly assess the motility capacity of TvVPS32-transfected parasites. To this end, TvEpNeo and TvVPS32 parasites were spotted onto soft agar, and their migration capacity was analyzed by measuring the size of the halo diameter from the inoculation point to the periphery of the plate. As shown in **Figure 11C**, the parasites transfected with TvVPS32 have a higher capacity of migration compared with parasites transfected with TvEpNeo, which might be suggesting a possible role for VPS32 protein in parasite motility.

## 4 Discussion

Flagella have been extensively described as important players for host invasion, pathogenicity, and intercellular communication in pathogenic protists, mainly in kinetoplastids (Frolov et al., 2018; Shimogawa et al., 2018; Kelly et al., 2020). However, the structural organization and biological functions of trichomonad flagella remain largely unexplored. Most of the studies about trichomonad flagella have focused on specializations of the flagellar membrane (Benchimol et al., 1982; Honigberg et al., 1984; Benchimol et al., 1992), propulsion force (Ribeiro et al., 2000; Lenaghan et al., 2014), and axoneme structure (Melkonian et al., 1991; Lopes et al., 2001; Lee et al., 2009). Here, we used a combination of electron microscopy techniques to reveal the ultrastructure of a novel EAS in *T. vaginalis* and *T. foetus*, the most studied and important human and veterinary trichomonads, respectively. Traditionally, it has been assumed that *T. vaginalis* and *T. foetus* do not have EASs (Melkonian et al., 1991; Benchimol, 2004; Rocha et al., 2010; Lenaghan et al., 2014); however, we observed, in addition to the classical axoneme, thin fibrillary structures surrounded by the flagellar membrane running longitudinally along the axonemes. This novel structure displays morphology of paraflagellar swellings when seen by SEM or light microscopy. These EASs are more frequently found at the tip of the AF and RF in *T. vaginalis* and *T. foetus*, respectively. Suggesting that EASs might be evolutionarily conserved in the Parabasalia phylum, the ultrastructural features of *T. vaginalis* and *T. foetus* EASs are similar to the extra-axonemal filaments described in other trichomonads and related parabasalid species, such as *Trichomitus batrachorum* (Mattern et al., 1973), *Tritrichomonas muris* (Viscogliosi and Brugerolle, 1993), *Pentatrichomonoides* sp. (Brugerolle et al., 1994), *Pseudotrypanosoma giganteum* (Brugerolle, 1999), and *Gigantomonas herculea* (Brugerolle, 2005).

Although the ultrastructure of *T. vaginalis* and *T. foetus* has been extensively investigated (Benchimol, 2004; de Andrade Rosa et al., 2013; de Souza and Attias, 2018), we believed there are some reasons that could explain why EASs had not been reported before. First, under axenic growth conditions, the EASs are only observed in 1%–11% of parasites. Considering these percentages, a careful observation under an electron microscope, mainly TEM, might be needed to be able to identify and properly investigate this structure. Second, as flagellar swellings can exhibit distinct morphologies, sizes, and relative positions, they may have been misinterpreted as a feature of cell death, i.e., flagellar blebbing, or an abnormality. Third, the EASs may have been considered as an artifact and just ignored or underappreciated by the investigators. In this regard, different authors using staining methods for light microscopy have described that the flagella of several parabasalids, including *T. vaginalis* and *T. foetus*, usually end with a granular or small swelling structure called “knob” (Kirby, 1951; Honigberg and King, 1964; Čepička et al., 2016); however, it has been suggested that “knobs” may be artifacts due to cell shrinkage during the fixation for protargol staining (Ceza et al., 2015; Čepička et al., 2016). Based on their location and morphologic similarities, we hypothesize that the EASs described here and the previously described “knobs” might be the same structure.

The EASs are found in the flagella of many cells including outer dense fibers and fibrous sheath of rodents and human sperm (Eddy et al., 2003; Linck et al., 2016), mastigonemes in *Chlamydomonas* (Liu et al., 2020), vane structures in the fornicate *Aduncisulcus paluster* (Yubuki et al., 2016), and the PFR of euglenoids and kinetoplastids (Zhang et al., 2021). They can run along the full length (outer dense fibers, fibrous sheath, and PFR), or just a portion, one- or two-thirds of the axoneme (mastigonemes and vane structures). All those EASs have a striated appearance when viewed using TEM, suggesting a regular high-order structure. Similarly, the *T. vaginalis* and *T. foetus* EAS has also a striated fibrillar structure; however, whereas the outer dense fibers, mastigonemes, and PFR are regular intricate structures, linked to the axoneme *via* outer microtubule doublets and found in all flagella from their respective cell types (Linck et al., 2016; Liu et al., 2020; Zhang et al., 2021), a) the trichomonad EAS is not observed in all cells and axonemes; b) it can be seen at the tip and/or middle of axoneme; c) no association between the extra-axonemal filaments and axoneme microtubule doublets is still found; and d) the organization and amount of the filaments can vary, resulting in two basic distinct morphologies, “sausage” and “spoon,” ranging in different sizes. Those findings indicate that the assembly of trichomonad EAS is not a regular feature and might require cell signaling responses. Additionally, our results suggest that the several shapes and sizes of trichomonad EAS might correspond to different phases of a single assembly event. We hypothesize that the process might start with a “sausage” EAS and the “spoon” morphology might be the “final destination” morphology. Further analysis by videomicroscopy could help us to confirm this hypothesis. Moreover, we do not know yet whether the trichomonad flagellar swellings are reversible and how they could be associated with flagellum assembly or disassembly processes. Importantly, the identification of non-regular and transient EASs has never been described. Additional studies are needed to investigate the assembly kinetics and protein composition of trichomonad EAS. The flagellar morphogenesis and assembly are still unknown in trichomonads, and further investigation is also necessary in this underresearched area.

The flagellum is a crucial host–pathogen interface, mediating the attachment of parasites to host tissues (Kelly et al., 2020). In this regard, EASs, such as PFR, might act as a flagellar support during tissue attachment in different stages of pathogens life cycle (Bastin et al., 1996; Maga and LeBowitz, 1999; Maharana et al., 2015). Also, this PFR has been proposed as a metabolic, homeostatic, regulatory, and sensory platform (Portman and Gull, 2010). These functions seem to be conserved among EASs during evolution. In this sense, the flagellar tip of *Crithidia fasciculata* is expanded up to six times its usual diameter upon contact with the insect host (Brooker, 1970). Also, arborescent outgrowths or “flagellipodia” were observed in the anterior flagellum of the bodonid flagellate *Cryptobia* sp. during their interaction to the snail *Triadopsis multilineata* (Current, 1980). Interestingly, the existence of flagellar morphological modifications seems to be related to adherence events along the life cycle in different flagellated organisms.

Here, we demonstrated that the EAS formation increases during the attachment process in *T. vaginalis* and *T. foetus*. This finding is relevant considering that these protozoans are extracellular organisms; thus, flagella and cell body are likely to play important roles in the initial adherence and survival of the pathogen on mucosal surfaces. It has been described that trichomonad flagella can interact with host epithelial cells, ECM proteins, yeasts, sperm cells, and bacteria (Casta e Silva Filho et al., 1988; Pereira-Neves and Benchimol, 2007; Midlej et al., 2009; Midlej and Benchimol, 2010). *Tritrichomonas foetus* uses the recurrent flagellum to establish the first contact upon attachment with the host cell (Singh et al., 1999). Here, we found that EASs are more frequent in the recurrent flagellum of *T. foetus*. Our results suggest a role of EASs during *T. vaginalis* and *T. foetus* attachment to the host cells as the EASs have been observed in direct contact with the epithelial cells and the network-shaped mesh of preputial mucus. Taking into account that *T. vaginalis* appears to use its flagella as the guiding end to migrate and penetrate host tissues (Kusdian et al., 2013), we consider that structural changes due to EASs by increasing the adhesion surface would also facilitate trichomonad displacement in a viscous environment (epithelial mucus) or some materials (e.g., semisolid media). However, future work is necessary to investigate this hypothesis.

In *T. vaginalis*, the EAS membranes possess high numbers of rosettes or intramembrane particles. The presence of intramembranous particles forming circular rosettes in the membrane of anterior flagellar of trichomonads has been previously reported (Benchimol et al., 1981; Benchimol and De Souza, 1990). The rosettes have been compared to particles involved in membrane fusion in *Tetrahymena* and hypothesized to contribute to active exo- and endocytosis (Satir et al., 1973; Lenaghan et al., 2014). These specialized integral membrane particles might be involved in active sensing of the environment and play a key role in controlling local calcium levels to regulate flagellar beating (Benchimol, 2004; Lenaghan et al., 2014). In this sense, the kinetoplastid PFR provides a platform for cAMP and calcium signaling pathways that control motility and host–pathogen interactions and for metabolic activities that may participate in energy transfer within the flagellum (Sugrue et al., 1988; Portman and Gull, 2010; Ginger et al., 2013; Shaw et al., 2019; Zhang et al., 2021). Similarly, the fibrous sheath of mammal sperm is a docking for key components in cAMP signaling pathways, implicated in the regulation of sperm motility (Eddy et al., 2003). Based on the role of EASs in other organisms and our results, a sensory role for EASs might be suggested in *T. vaginalis*. The higher surface area of flagellar swellings due to EASs may provide a site for a greater number of rosettes.

The flagellar surface is a highly specialized subdomain of the plasma membrane, and flagellar membrane proteins are key players for all the biologically important roles of flagella (Landfear et al., 2015). In this sense, flagella are emerging as key players in cell-to-cell communication *via* shedding of MVs. MVs are observed protruding from flagellar tips of mammal cells (Nager et al., 2017; Salinas et al., 2017), the nematode *Caenorhabditis elegans* (Wang and Barr, 2018), and protists, including *Chlamydomonas* (Long et al., 2016), *Trypanosoma brucei* (Szempruch et al., 2016), and *T. vaginalis* (Nievas et al., 2018), suggesting that flagella may support MV biogenesis. Here, we found MV-like structures protruding from the trichomonad EASs. A higher area and curvature of the flagellar swellings may provide an advantage for the flagella to be used as a subcellular location for MV biogenesis. In this context, ESCRT is an important mechanism known to facilitate the outward budding of the membrane.

ESCRT proteins are emerging as a versatile membrane scission machine that shapes the behavior of membranes throughout the cell. In *Chlamydomonas reinhardtii*, ESCRT components are found in isolated ciliary transition zones, ciliary membranes, and ciliary microvesicles (Long et al., 2016). Additionally, ESCRT proteins mediate MV release and influence flagellar shortening and mating (Diener et al., 2015; Long et al., 2016). ESCRT proteins are also found at the base of sensory cilia of *C. elegans* (Hu et al., 2007), suggesting that the ESCRT machinery is involved in flagellar function. In addition to mediating membrane budding and flagellar MV shedding, ESCRT components may act as sensors for the generation and stabilization of the membrane curvature of flagella (Long et al., 2016; Wang and Barr, 2018; Jung et al., 2020). Consistent with this, silencing of Vps36 in trypanosomes, an ESCRT component, compromised the secretion of exosomes (Eliaz et al., 2017). In *T. vaginalis*, VPS32 protein (a member of the ESCRT-III complex) has been identified in the proteomic analyses of isolated exosomes and MVs (Twu et al., 2013; Nievas et al., 2018). In *T. foetus*, our group previously reported that VPS32 is localized on cytoplasmic vesicles, and a redistribution of the protein to the midbody is observed during the cellular division, indicating a role of this protein in controlling mitosis (Iriarte et al., 2018). Here, besides the cytosolic vesicles, we revealed that VPS32 is present in the surface as well as in MVs protruding from EASs, in both *T. foetus* and *T. vaginalis*. Specifically, ESCRT-III has been shown to be crucial for diverse membrane remodeling events, the pinching off and release of MVs (Huber et al., 2020). Interestingly, we observed that the formation of paraflagellar swellings, the adhesion to fibronectin-coated coverslips, and the percentage of cells with MVs on the EASs increased in parasites overexpressing VPS32; however, further analysis is needed to investigate whether the ESCRT-III complex might be involved in EAS formation and host–parasite interactions. Based on the function of the ESCRT-III complex in other organisms, we could speculate that VPS32 might be regulating the dynamic flagellar membrane transformation that occurs during EAS formation. Alternatively, VPS32 could participate in the biogenesis and final scission necessary for MV release from the flagellar EAS membranes and subsequent membrane repair. Importantly, to our knowledge, this is the first identification of an ESCRT protein associated with the flagella of a pathogenic protist.

In addition to the release of extracellular vesicles, the contact between cells is also an important event in cell communication. Trypanosomes can interact with each other by flagellar membrane fusion, which could be partial and transient or irreversible and along the entire length of the flagellum (Imhof et al., 2016). These membrane fusion events might represent an alternative bidirectional mechanism used for protein exchange with other individuals in a population. Fusion between membrane flagellar has been reported in *C. fasciculata* (Brooker, 1970), *Leptomonas lygaei* (Tieszen et al., 1989), and *Trypanosoma melophagium* (Molyneux, 1975). Curiously, in *C. fasciculata*, the existence of interflagellar type B desmosomes (temporary structures) between adjacent flagella of microorganisms in contact with each other has been described. Such junctions appear to maintain the “cluster” integrity that this protist forms in the gut of the mosquito or in cultures (Brooker, 1970). The association of “clustering” and amoeboid transformation with a higher parasite adherence capacity has been reported in *T. vaginalis*; however, the mechanisms behind this phenomenon still remain unknown (Lustig et al., 2013). Here, we demonstrated that trichomonads can connect with each other by EAS flagella, suggesting that this connection could contribute to cell communication. Supporting this, we observed that adhesion assays with Alcian blue- and fibronectin-coated coverslips induced amoeboid transformation and cell clusters (only Alcian blue) and increased the EAS formation, suggesting a positive correlation between amoeboid transformation, cells clusters, and EAS formation.

The results obtained here also demonstrated that TvVPS32 is present in the EAS of parasites in contact with each other, and interestingly, parasites overexpressing TvVPS32 showed greater motility in semisolid agar. Previously, we analyzed the growth rates of TvEpNeo and TvVPS32 parasites and we did not observe significant differences (data not shown); thus, an increase in halo size diameter could be related to migration and not with increased parasite number. In trypanosomatids and euglenoids, the PFR is required for shaping the flagellar beat, acting as a biomechanical structure that supports the non-planar motility (Cicconofri et al., 2021; Zhang et al., 2021). It has been reported that *T. brucei* engages polarized migrations across the semisolid agarose surface mediated by flagellum communication (Oberholzer et al., 2010). The trichomonad flagella have multiply flagellar-beating motions, similar to the “run and tumble” mechanism observed in *Chlamydomonas*, where cells oscillate between nearly straight swimming and abrupt large reorientations (Lenaghan et al., 2014; Tung et al., 2015). However, the mechanical biocomponents responsible for generating multiple waveforms in the trichomonad flagella are still unknown. Taking into account that VPS32 is the scission effector in different cellular membranes (Tang et al., 2015), we could speculate that this protein might be responsible for regulating different scission events during parasite:parasite communication or participating in flagellar membrane transformation important for parasite motility. In this sense, although the plate assay is an indirect measure, these results might be suggesting a role of the EAS in parasite motility. However, future studies are needed to establish the specific function of ESCRT-III and EAS within this process in trichomonads.

This study will certainly shed light to our understanding on the flagella biology in pathogenic trichomonads. In summary, we described a novel EAS that provides a larger flagellar contact surface and added to this, the presence of rosettes and MVs in their membranes leads us to speculate that these structures could be involved in sensing, signaling, cell communication, and pathogenesis in trichomonads. In the future, continuing studies about the structure, proteomic, and assembly of EASs will enable us to better define how those mentioned functions are mediated by flagella in these extracellular parasites. Because the flagellum is an essential organelle, defining the flagellar morphology and roles in *T. vaginalis* and *T. foetus* may therefore help us to understand how the parasite colonizes the urogenital tract and how to prevent or treat infections and to uncover novel drug targets. In addition, trichomonads could emerge as a model system for studies of the conserved aspects of eukaryotic flagellum and EASs, providing new insights into evolutionary and functional aspects with direct relevance to other eukaryotes, including humans, in which flagella/cilia are essential for development and physiology, and defects can provoke several morbidities or fatal diseases.

## Data Availability Statement

The original contributions presented in the study are included in the article/**Supplementary Material**. Further inquiries can be directed to the corresponding authors.

## Author Contributions

Conceived and designed the experiments: VC, NM, and AP-N. Performed the experiments: VC, LI, AM-M, TA, and AP-N. Analyzed the data: VC, NM, and AP-N. Contributed reagents/materials/analysis tools: VC, NM, and AP-N. Wrote the paper: VC, NM, and AP-N. All the authors were involved in reviewing and editing the manuscript. All authors contributed to the article and approved the submitted version.

## Funding

This work was supported by Conselho Nacional de Desenvolvimento Científico e Tecnológico (CNPq; grants 404935/2016-8 and 400740/2019-2 to AP-N) and by ANPCyT (grant BID PICT 2016-0357-VC to VC).

## Conflict of Interest

The authors declare that the research was conducted in the absence of any commercial or financial relationships that could be construed as a potential conflict of interest.

## Publisher’s Note

All claims expressed in this article are solely those of the authors and do not necessarily represent those of their affiliated organizations, or those of the publisher, the editors and the reviewers. Any product that may be evaluated in this article, or claim that may be made by its manufacturer, is not guaranteed or endorsed by the publisher.
