# Supplementary material for: Ultrastructural and Functional Analysis of a Novel Extra-Axonemal Structure in Parasitic Trichomonads
Source: Front Cell Infect Microbiol. 2021 Nov 9;11:757185. doi: 10.3389/fcimb.2021.757185 (PMC8630684; doi:10.3389/fcimb.2021.757185)
Supplement: Supplementary file 1 [file DataSheet_1.docx]

***Supplementary Material***

1. **Supplementary Figures**

**Supplementary Figure 1.** SEM of flagellar “sausage-like” swelling in *T. vaginalis*. (**A**) The swellings can be seen laterally (yellow arrow) to or surrounding (white arrows) the flagellum. (**B**) The swellings display a range size from 0.1 to 1 µm in thickness and a length from 0.3 to 6 µm. AF, anterior flagella; RF, recurrent flagellum.

**Supplementary Figure 2.** SEM of flagellar “spoon-like” swelling. The flagellum (F) folds around the swelling (*), forming a rounded or ellipsoid structure with a range size from 0.5 to 2.5 µm in the major axis in *T. foetus* (**A, B**) and more than 4 µm long in *T. vaginalis* (**C**). (**D, E**) Frontal views. The “spoon-like” structure exhibits a flattened (**D**) or concave (arrows) surface (**E**). (**F, G, H**) Side views. The structure (arrowheads) displays an aligned (**F**), curved (**G**), or convex (**H**) appearance.

**Supplementary Figure 3.** Ultrastructure of the “sausage-like” swelling at the tip of *T. vaginalis* flagella by different perspectives. First row, SEM (**A**), negative staining (**B**) and ultrathin section (**C)** of swellings in a bottom view. Second row, SEM (**D**) and negative staining (**E, F**) of structure in a top view. Third row, SEM (G) and negative staining (H) of swelling in an oblique view. The dotted lines indicate boundary between axoneme (Ax) and the extra-axonemal filaments (*). In a SEM bottom view (A), notice that swelling (*) partially surround the flagellum (F), whereas in a top view (D) seems that the flagellum is totally surrounded by the swelling. In an oblique view (G-H), observe that the axoneme is in a slit of the swelling.

**Supplementary Figure 4.** Fine structure of the “sausage-like” swelling in the middle of *T. vaginalis* flagella. (**A**) SEM. (**B**) Negative staining. (**C**) Ultrathin section. (**B, C**) The structure is formed by thin extra-axonemal filaments (*) that run longitudinally along the axoneme (Ax). The dotted lines indicate boundary between axoneme and the extra-axonemal filaments. F, flagellum.

**Supplementary Figure 5.** Ultrastructure of the sausage shaped swelling at the tip of *T. vaginalis* recurrent flagellum. (**A**) SEM. (**B**) Transversal and (**C**) cross sections. (**B, C**) The structure is formed by thin extra-axonemal filaments (*). RF, recurrent flagellum; UM, undulating membrane; Ax, axoneme.

**Supplementary Figure 6.** SEM of flagellar swellings in the recurrent flagellum (RF) of *T. vaginalis* **(A, C, E)** and *T. foetus* **(B, D, F)**. Notice that the *T. vaginalis*-RF has no free portion and exhibits sausage shaped swellings (arrows) of different sizes. Both sausage (**B**) and spoon (**D, F**) shaped swellings are seen in the free tip of *T. foetus*-RF. UM, undulating membrane.

**Supplementary Figure 7.** The EASs formation increase during trichomonads attachment on Alcian blue-coated coverslips. (**A**) Representative SEM micrographs of *T. vaginalis* and *T. foetus* after the adhesion assay. Control: parasites incubated on uncovered coverslips in humidity chamber for 0.5 h at 37ºC, collected with a pipette, harvested by centrifugation, and prepared for SEM; Attached and non-attached: parasites incubated on 1% Alcian blue-coated glass coverslips in humidity chamber for 0.5 h at 37ºC and rigorously washed with PBS to remove non-attached cells. Attached parasites remain on the coverslips even after several washes. Non-attached parasites were collected with a pipette, harvested by centrifugation, and prepared for SEM. “Control” is formed by non-adherent, suspended cells from uncovered coverslips, whereas non-adherent parasites from fibronectin are called “Non-attached”. In Control, the parasites display the typical pyriform cell body and no cell clusters. In Attached and Non-attached group, the cells are clustered, exhibiting an amoeboid or ellipsoid form and many flagellar swellings (arrows). AF, anterior flagella; RF, recurrent flagellum; UM, undulating membrane. (**B, C**) Quantitative analysis of the percentage of *T. vaginalis* (**B**) and *T. foetus* (**C**) with and without swelling after the adhesion assay. Three independent experiments in duplicate were performed and 500 parasites were randomly counted per sample using SEM. Data are expressed as percentage of parasites ± SD. The percentage of parasites displaying flagellar swelling in the Alcian blue-attached group is higher s when compared to control. Unexpectedly, the percentage of *T. vaginalis* with EAS in the Alcian blue-Non-attached group was significantly higher when compared to control. * p<0.05; ** p<0.01; *** p<0.001 compared to control using One-Way ANOVA test (Kruskal-Wallis test; Dunn’s multiple comparisons test).

**Supplementary Figure 8.** SEM *T. vaginalis* after host cell interaction. HeLa cells were co-incubated with *T. vaginalis* at cell ratios of 1:1 or 5:1 parasite:host cell in PBS-F (PBS with 1% FBS at pH 6.5) at 37°C for 30 min. Flagellar swelling (arrows) are seen in some parasites (P). Notice that some swellings are in direct contact to the host cells (H).

**Supplementary Figure 9.** SEM of flagellar swellings in *T. vaginalis* and *T. foetus*. (**A-B**) Flagellar swelling (arrow) in a *T. vaginalis* (P) attached on a prostatic epithelial cell (H). (**C, D**, **G, H**) Swellings (arrows) in direct contact to bacteria (B) after host cells interaction assays. (**E, F**) Swellings (arrows) in *T. foetus* (P) adhered to the network-shaped mesh of preputial mucus. AF, anterior flagella; RF, recurrent flagellum.

**Supplementary Figure 10.** Immunogold of VPS32-HA transfected *T. vaginalis*. (**A-B**) Lower magnification and insets of non-extra-axonemal regions of the same flagella in the Fig. 10B. Few gold particles (arrows) are seen in the flagellar area without extra-axonemal structure. Asterisks, extra-axonemal structure. (**C**) General and detailed views of three anterior flagella (Ax1, Ax2 and Ax3). The tip of the second flagella (Ax2) displays an extra-axonemal structure (*) with an intense labeling (arrows). No gold particles are noticed in the flagella and regions without extra-axonemal structures. (**D**) Gold particles (arrows) are seen in the surface of a spoon shaped extra-axonemal structure. The dotted lines indicate boundary between axoneme (Ax) and the extra-axonemal filaments (*). No labelling is seen in the non-EAS region.

**Supplementary Figure 11.** (**A**) Representative SEM micrographs of MVs-like structures (arrowheads) protruding from the flagellar membrane of the EASs (*) of VPS32-transfected *T. vaginalis* and *T. foetus*. (**B**) % of EASs with protruding MVs on their surface. Three independent experiments in duplicate were performed and 100 parasites exhibiting at least one swelling were randomly counted per sample using SEM. Data are expressed as means ± SD. The percentage of VPS32-overexpressing parasites with MVs on the EASs is 1.6-fold higher when compared to control cells (EpNeo - empty plasmid transfected). **p<0.01 compared to control using One-Way ANOVA test (Kruskal-Wallis test; Dunn’s multiple comparisons test). (**C**) Quantitative analyses of transfected *T. vaginalis* and *T. foetus* after adhesion assay on fibronectin-coated coverslips**.** Fibronectin-coated coverslips were prepared by first covering them with 100 µL fibronectin (Sigma F0556: working solution of 10 µg/mL in sterile PBS) for 1h at room temperature and washing them with sterile PBS. Parasites (1x10^6^ cells/mL) were washed in PBS (pH 7.2) and resuspended in PBS.  A suspension of 50 µL was incubated on fibronectin-coated glass coverslips in humidity chamber for 2 h at 37ºC. Next, the coverslips were rigorously washed with PBS to remove non-adherent parasites. The parasites adhesion was monitored using an inverted phase contrast microscope. Three independent experiments were performed, and parasites were randomly counted in 50 fields using a light microscopy. Data are expressed as means ± SD.  Error bars represent standard deviations and asterisks denote statistically significant differences determined by One-Way ANOVA test (Kruskal-Wallis test; Dunn’s multiple comparisons test). (p value=0,0005). The number of VPS32-overexpressing parasites per field after adhesion assays is 2.4-fold higher when compared to control parasites (EpNeo) (**D**) Representative western blot using an anti-HA antibody. The antibody reacted specifically with a band near 24-kDa in transfected parasites, indicating the expression of VPS32 protein. 5 X 10^7^ parasites (TvVPS32 and TfVPS32) were used.
