# Supplementary figures and images for: Ultrastructural and Functional Analysis of a Novel Extra-Axonemal Structure in Parasitic Trichomonads
Source: Front Cell Infect Microbiol. 2021 Nov 9;11:757185. doi: 10.3389/fcimb.2021.757185 (PMC8630684; doi:10.3389/fcimb.2021.757185)

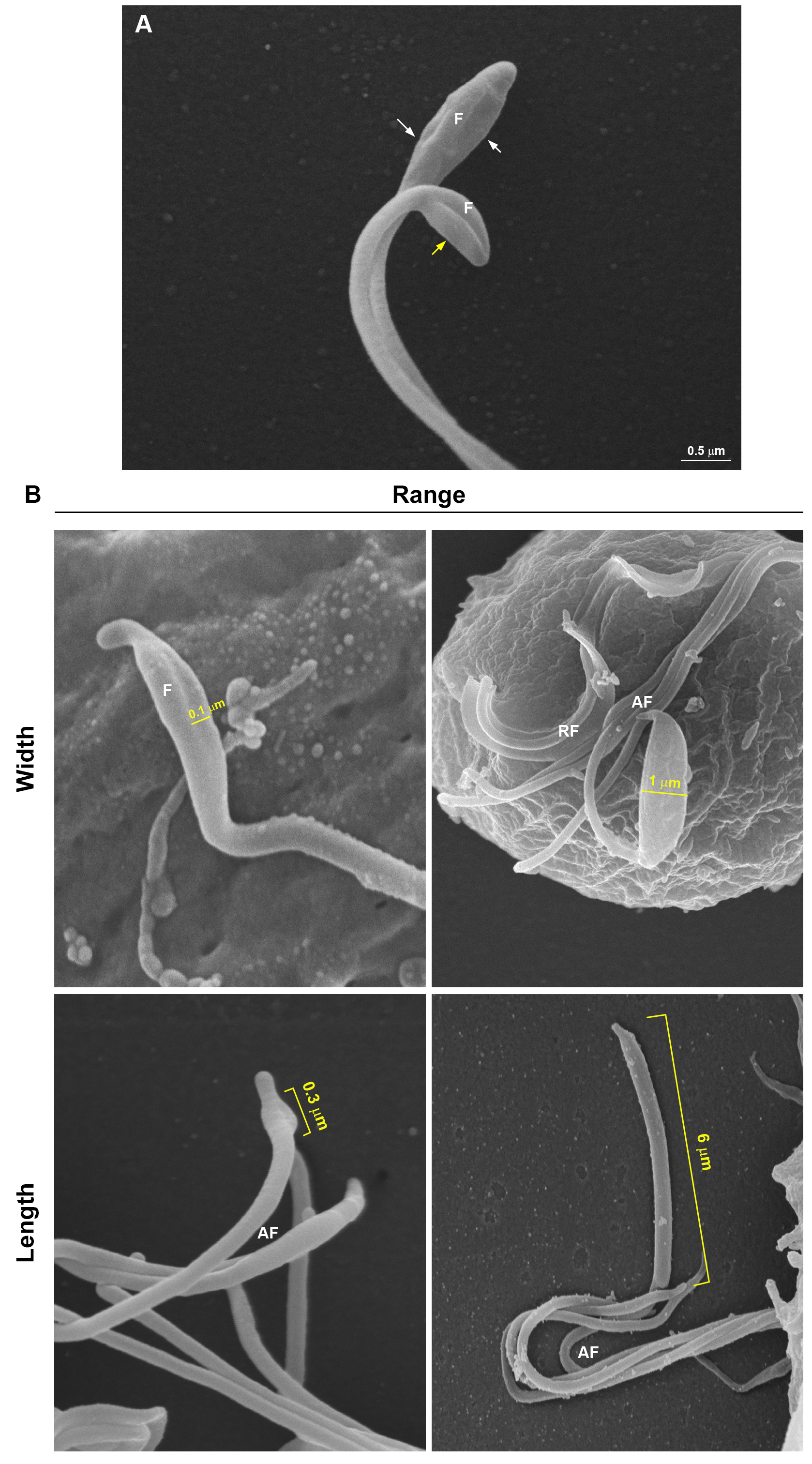

Supplement: Supplementary file 2 [file Image_1.tif]

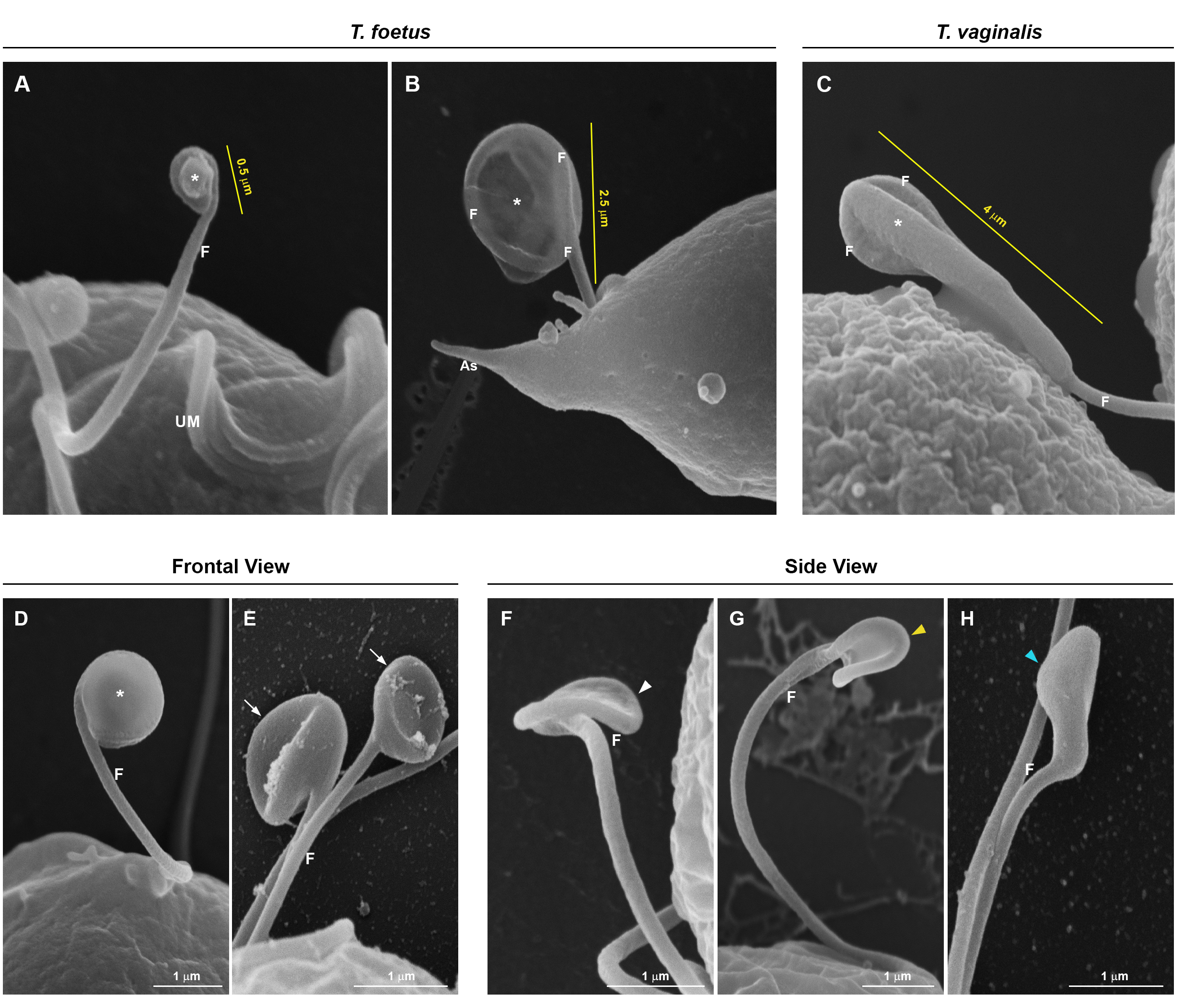

Supplement: Supplementary file 3 [file Image_2.tif]

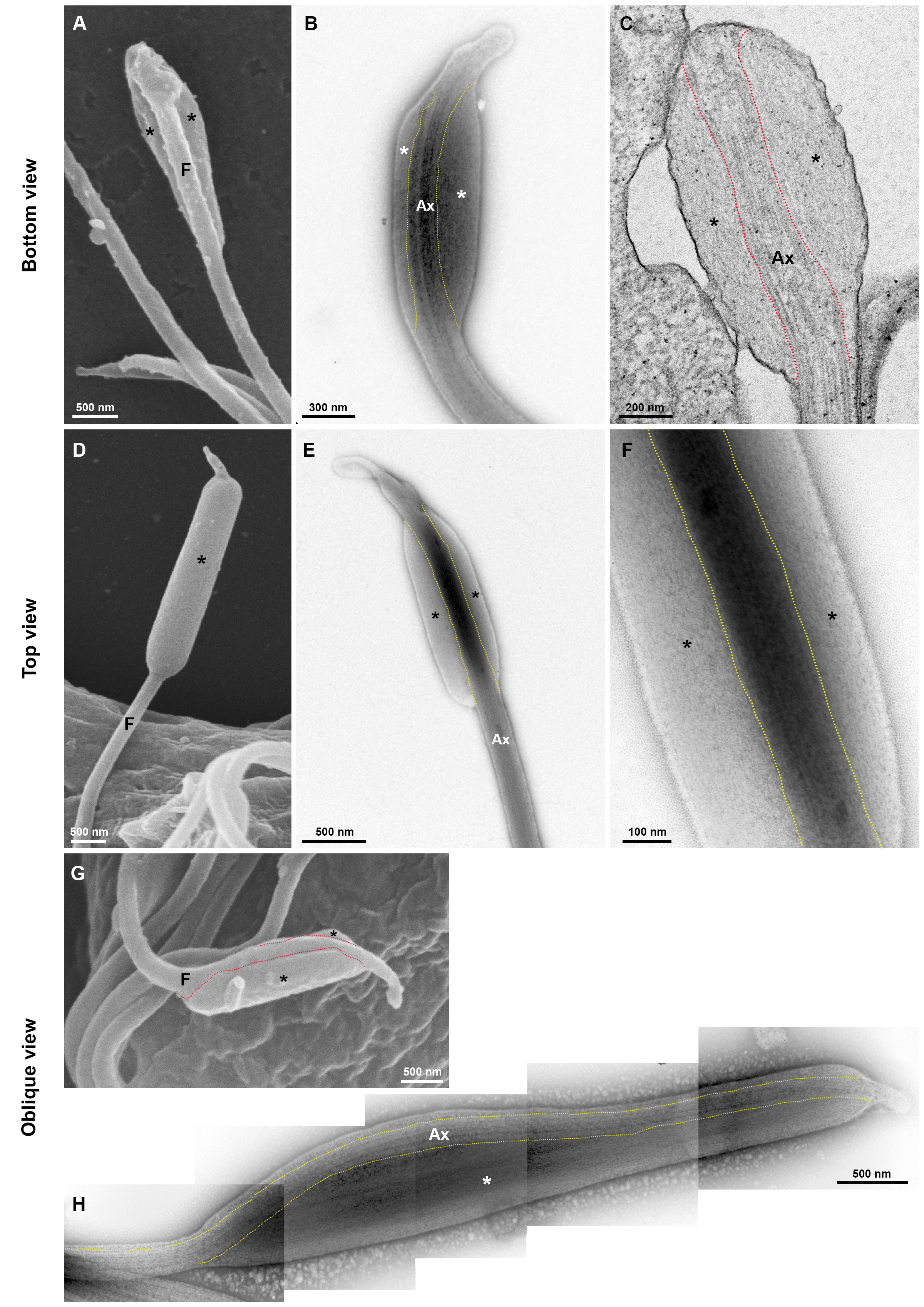

Supplement: Supplementary file 4 [file Image_3.tif]

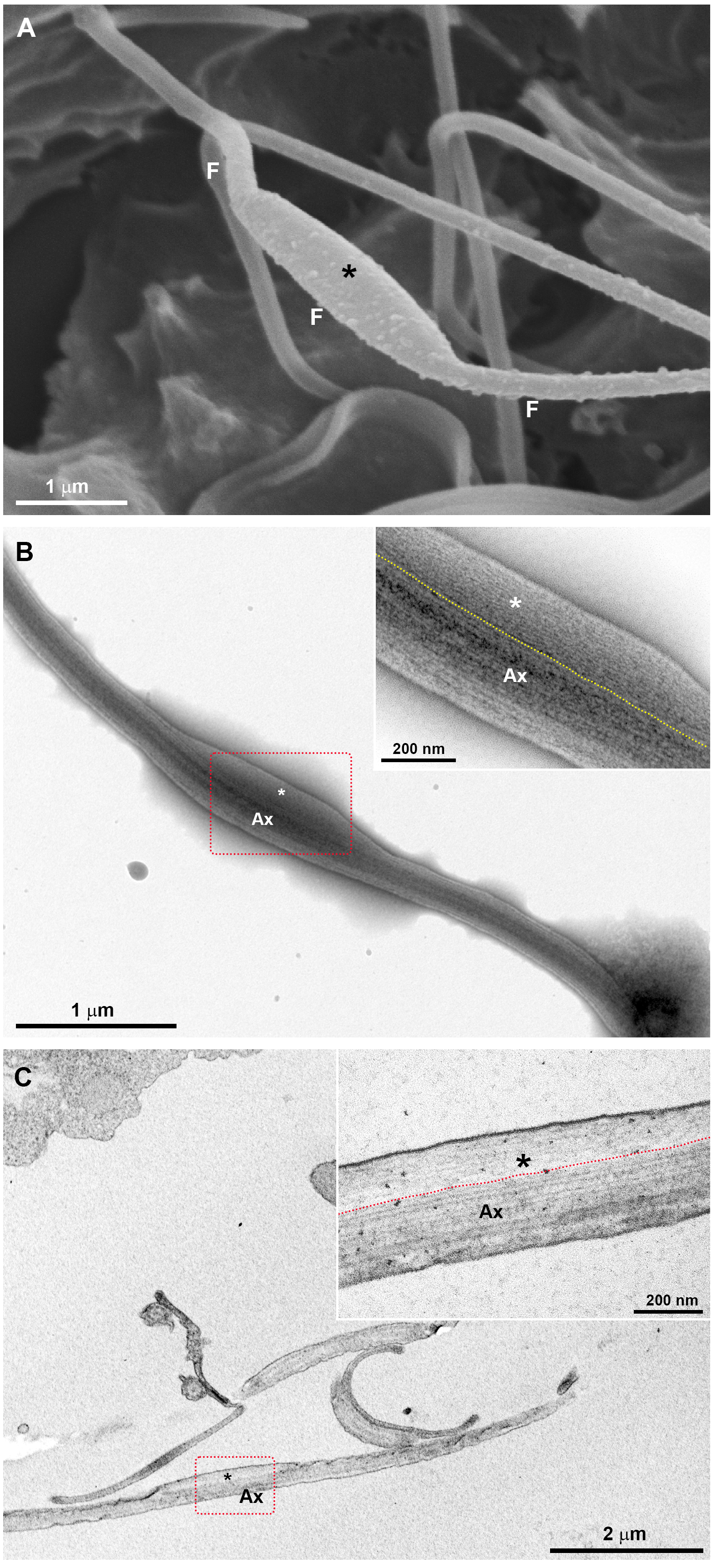

Supplement: Supplementary file 5 [file Image_4.tif]

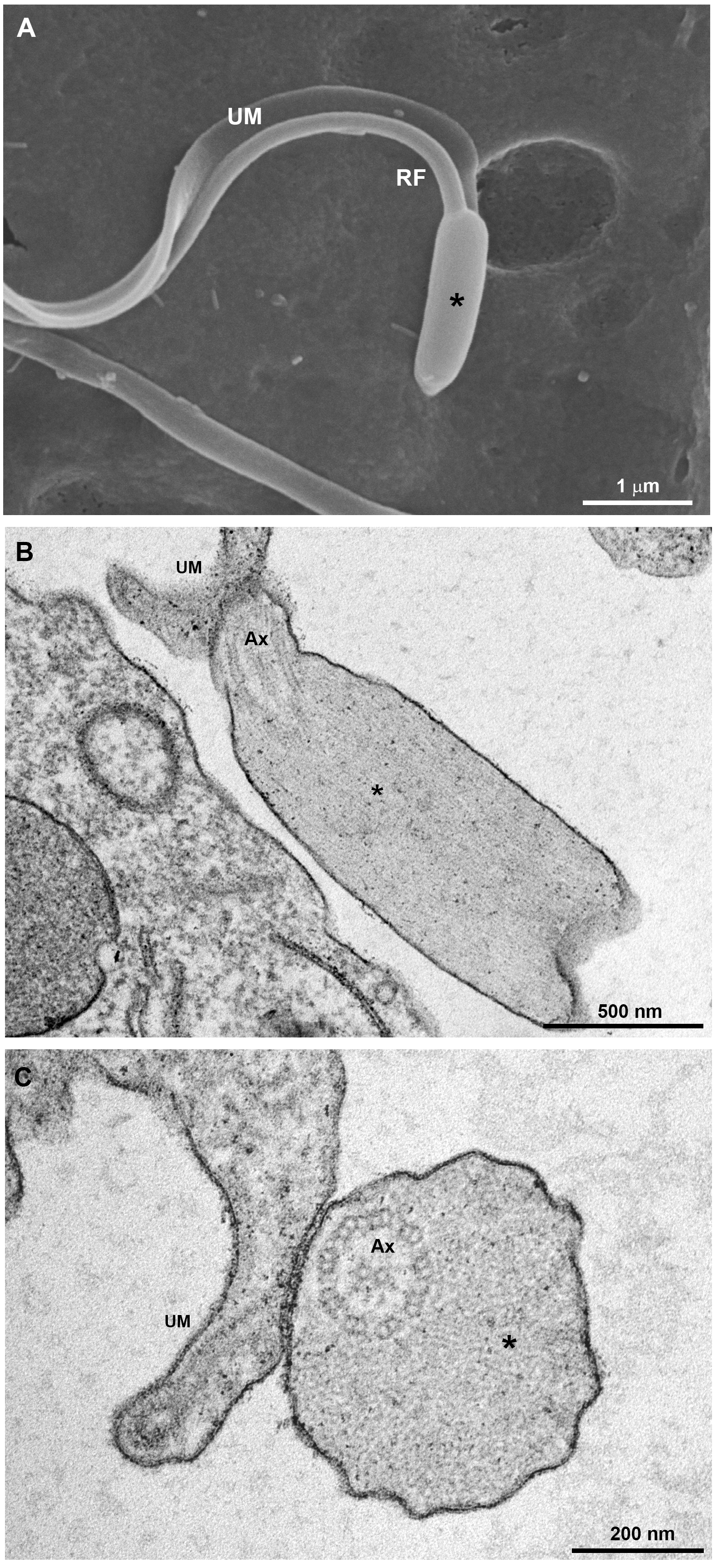

Supplement: Supplementary file 6 [file Image_5.tif]

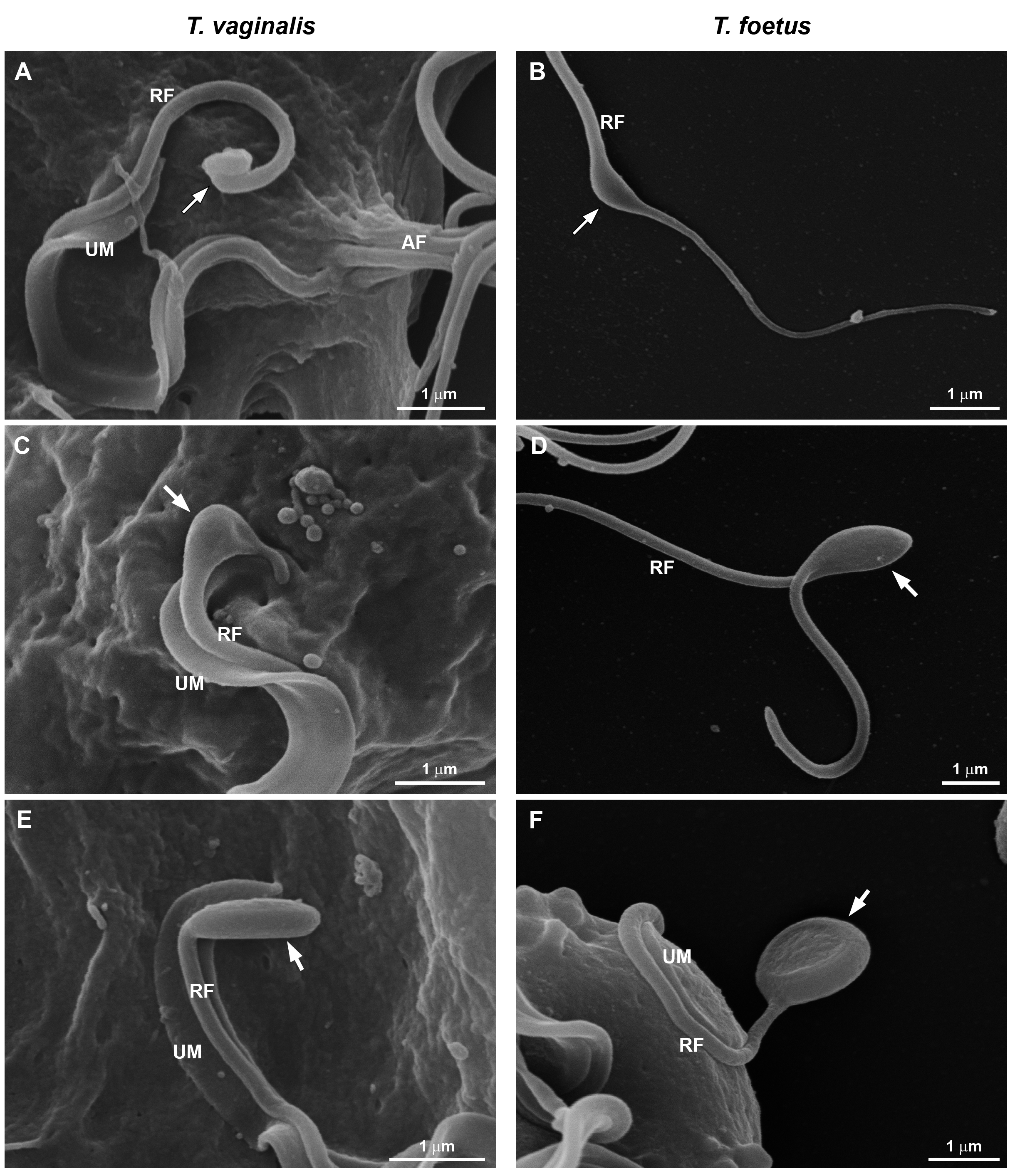

Supplement: Supplementary file 7 [file Image_6.tif]

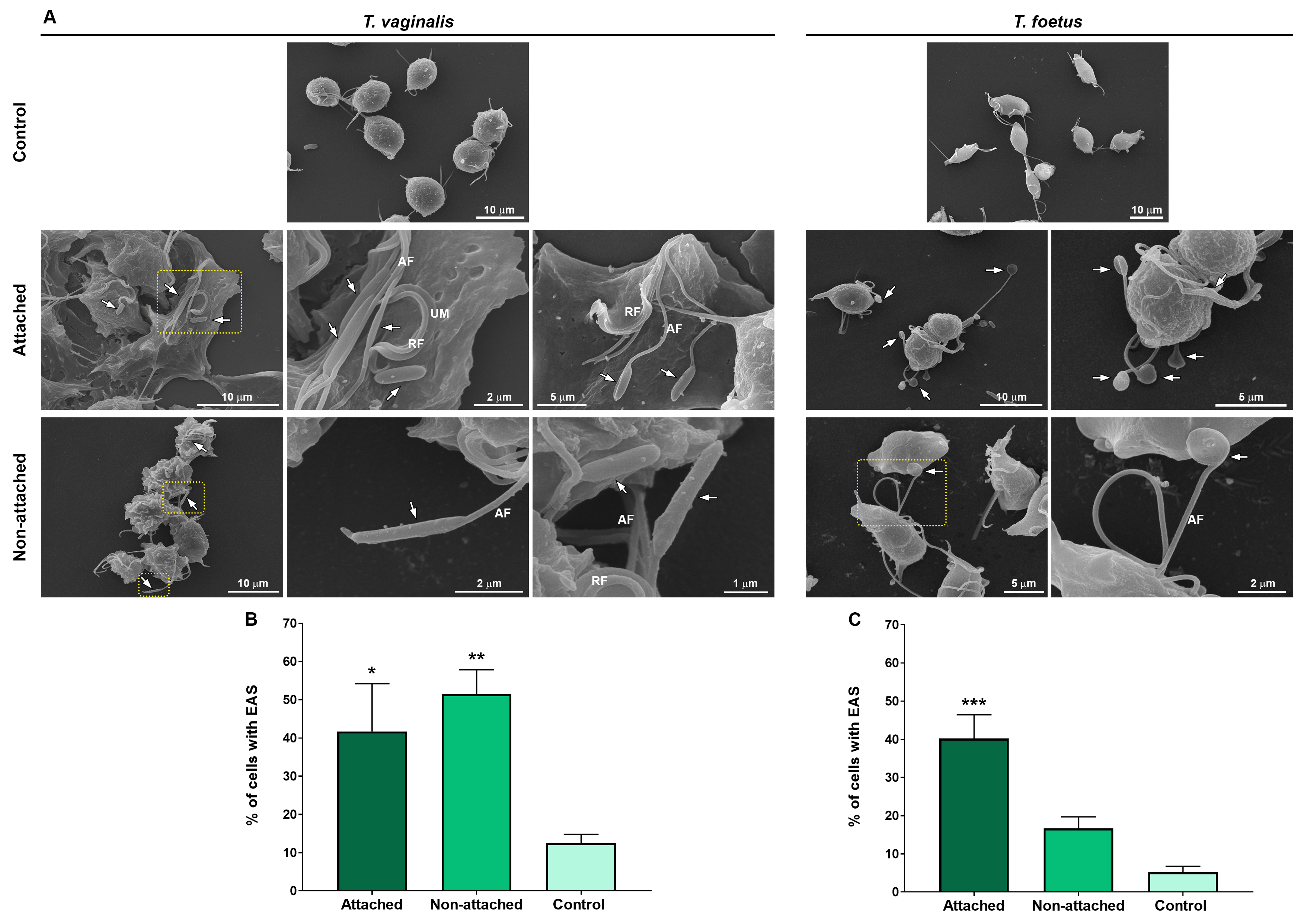

Supplement: Supplementary file 8 [file Image_7.tif]

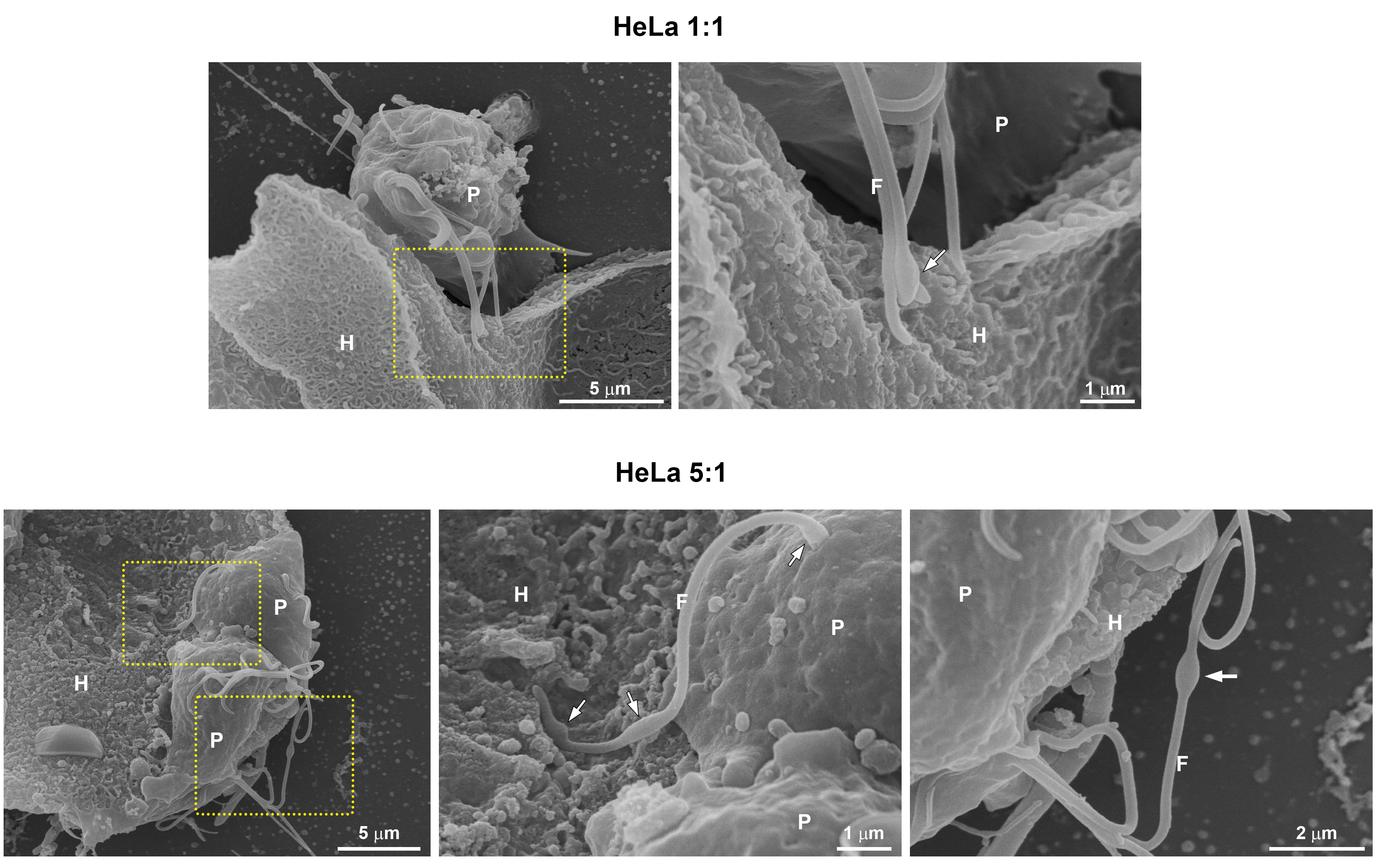

Supplement: Supplementary file 9 [file Image_8.tif]

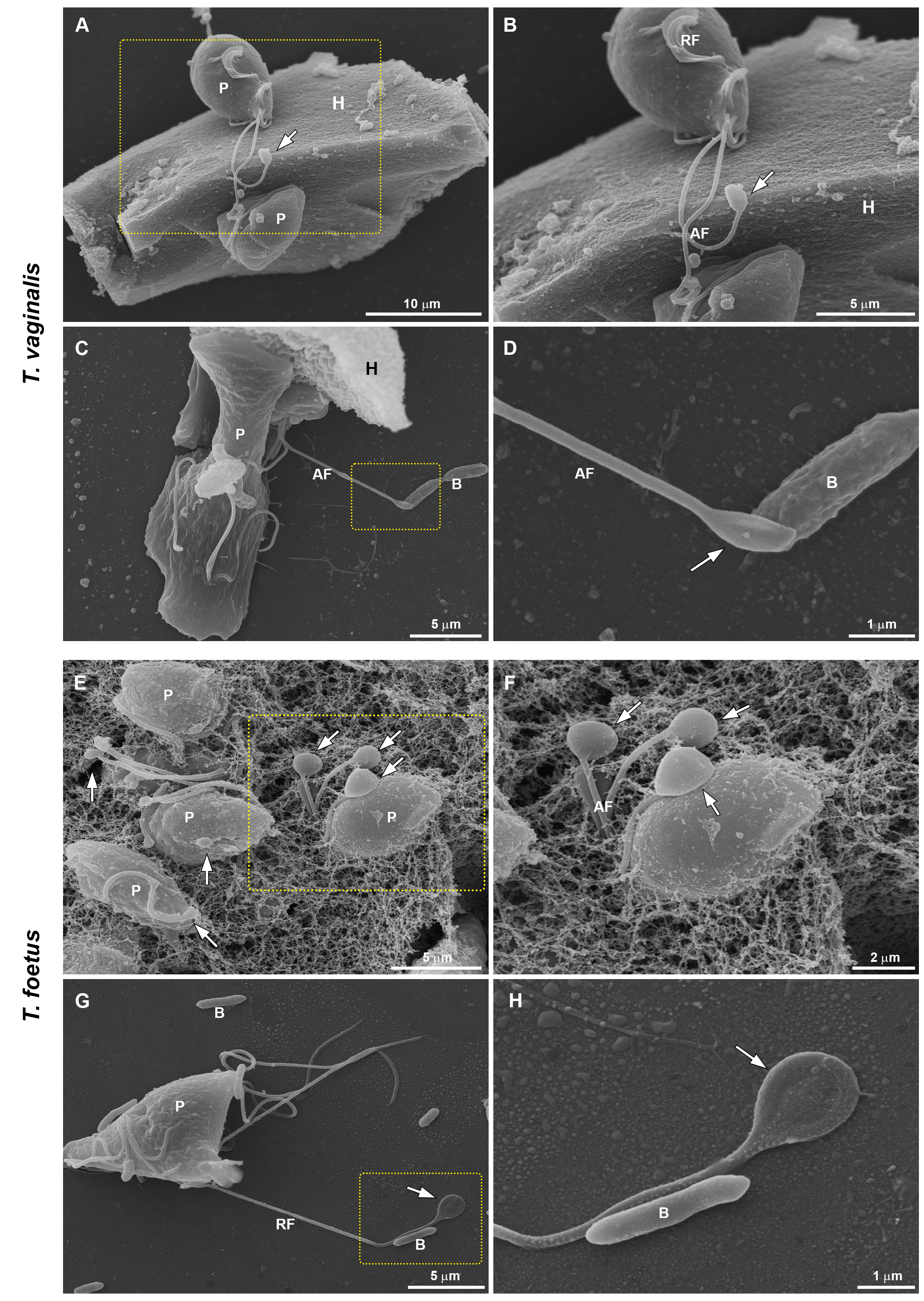

Supplement: Supplementary file 10 [file Image_9.tif]

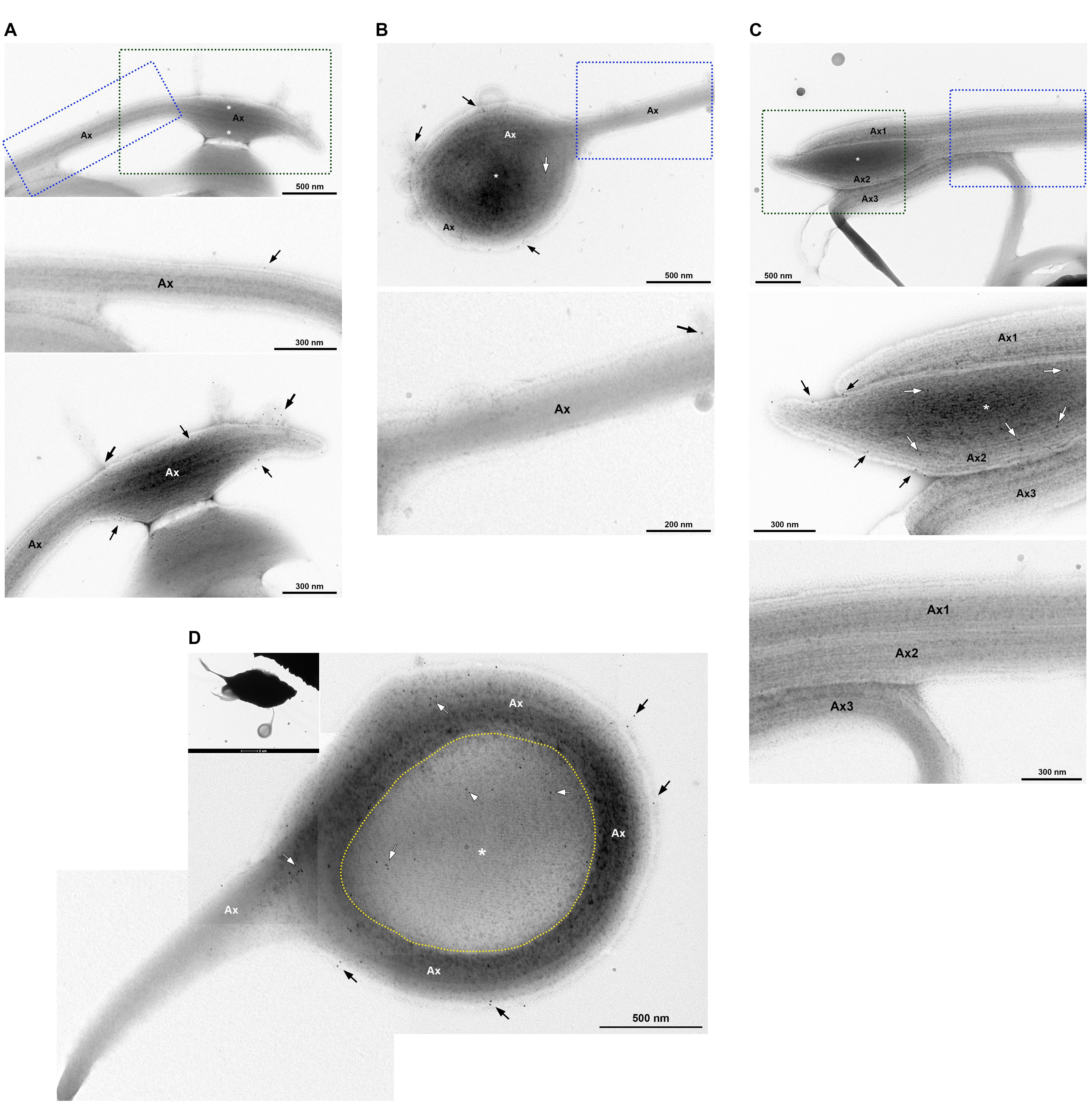

Supplement: Supplementary file 11 [file Image_10.tif]

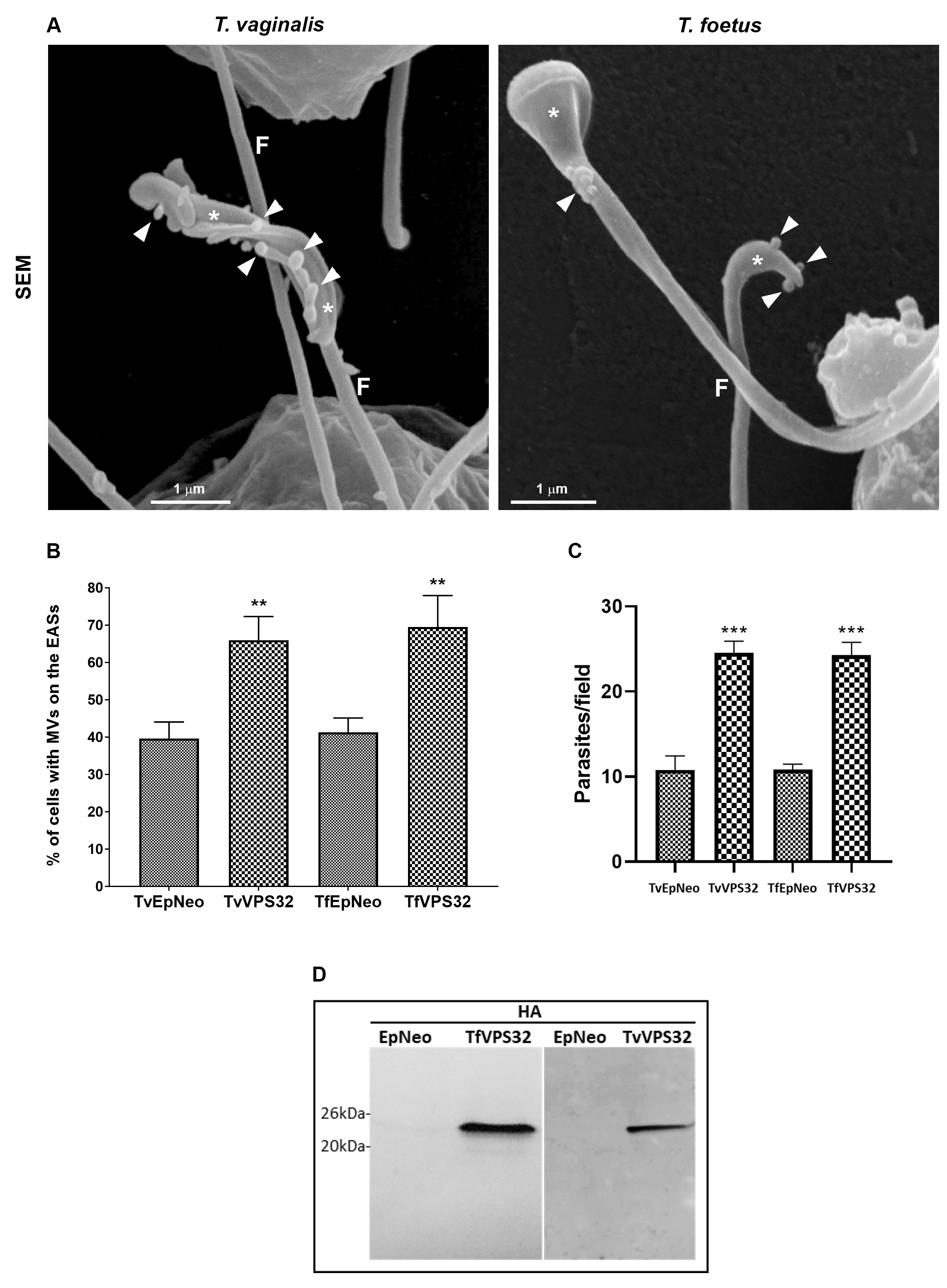

Supplement: Supplementary file 12 [file Image_11.tif]
